# Supplementary material for: Transcriptome analysis and identification of genes associated with floral transition and fruit development in rabbiteye blueberry (Vaccinium ashei)
Source: PLoS One. 2021 Oct 28;16(10):e0259119. doi: 10.1371/journal.pone.0259119 (PMC8553168; doi:10.1371/journal.pone.0259119)

PLOS ONE

Transcriptome analysis of genes associated with flowering and fruit set in rabbiteye blueberry (Vaccinium ashei)

--Manuscript Draft--

**Manuscript Number:**	PONE-D-21-18707
**Article Type:**	Research Article
**Full Title:**	Transcriptome analysis of genes associated with flowering and fruit set in rabbiteye blueberry (Vaccinium ashei)
**Short Title:**	Transcriptome analysis of genes associated with flowering and fruit set in rabbiteye blueberry
**Corresponding Author:**	xuan gao<br>Anhui Normal University<br>WuHu, Anhui CHINA
**Keywords:**	blueberry; transcriptome; flowering; fruit; phytohormone
**Abstract:**	Flowering and fruit set are important traits affecting fruit quality and yield in rabbiteye blueberry (Vaccinium ashei). Intense efforts have been made to elucidate the influence of vernalization and phytohormones on flowering, but the molecular mechanisms of flowering and fruit set from vegetative growth to reproductive development remain unclear. We divided flowering and fruit set into leaf bud (S1), flower bud (S2), initial flower (S3), bloom flower (S4), pad fruit (S5), and cup fruit (S6) based on phenotype and identified 7, 1338, 69, 657, and 187 unique differentially expressed genes (DEGs) in comparisons of S2/S1, S3/S2, S4/S3, S5/S4, and S6/S5, respectively. There were obviously more DEGs in S3/S2 and S5/S4 than in S2/S1, S4/S3, and S6/S5, suggesting that S3/S2 and S5/S4 represent major transitions from buds to fruit in blueberry. Gene ontology enrichment analysis indicated these DEGs were mostly enriched in phytohormone, transporter protein, and transcription factor categories. Gibberellic acid was important in early reproduction, while the expression of auxin response factors increased throughout the process from bud to fruit development. Transporter proteins were mainly associated with minerals in the early stage and sugars in later developmental stages. MYB86 expression was up-regulated in both flower development and initial fruit development, suggesting multiple functions in blueberry. Our study provides deep insights into the molecular mechanism underlying flowering and fruit set of rabbiteye blueberry.
**Order of Authors:**	xuan gao
	Lida wang
	Hong Zhang
	Bo Zhu
	Guosheng lv
	Jiaxin Xiao
**Additional Information:**	
**Question**	**Response**
**Financial Disclosure**<br><br>Enter a financial disclosure statement that describes the sources of funding for the work included in this submission. Review the submission guidelines for detailed requirements. View published research articles from *PLOS ONE* for specific examples.	The funders had no role in study design, data collection and analysis, decision to publish, or preparation of the manuscript.

Powered by Editorial Manager® and ProduXion Manager® from Aries Systems Corporation

This statement is required for submission and **will appear in the published article** if the submission is accepted. Please make sure it is accurate.<br><br>**Unfunded studies**<br>Enter: *The author(s) received no specific funding for this work.*<br><br>**Funded studies**<br>Enter a statement with the following details:<br>• Initials of the authors who received each award<br>• Grant numbers awarded to each author<br>• The full name of each funder<br>• URL of each funder website<br>• Did the sponsors or funders play any role in the study design, data collection and analysis, decision to publish, or preparation of the manuscript?<br>• **NO** - Include this sentence at the end of your statement: *The funders had no role in study design, data collection and analysis, decision to publish, or preparation of the manuscript.*<br>• **YES** - Specify the role(s) played.<br><br>* typeset	
**Competing Interests**<br><br>Use the instructions below to enter a competing interest statement for this submission. On behalf of all authors, disclose any competing interests that could be perceived to bias this work—acknowledging all financial support and any other relevant financial or non-financial competing interests.<br><br>This statement is required for submission and **will appear in the published article** if the submission is accepted. Please make sure it is accurate and that any funding sources listed in your Funding Information later in the submission form are also declared in your Financial Disclosure statement.<br><br>View published research articles from	The authors have declared that no competing interests exist.

Powered by Editorial Manager® and ProduXion Manager® from Aries Systems Corporation

PLOS ONE for specific examples.<br><br>**NO authors have competing interests**<br><br>Enter: *The authors have declared that no competing interests exist.*<br><br>**Authors with competing interests**<br><br>Enter competing interest details beginning with this statement:<br><br>*I have read the journal's policy and the authors of this manuscript have the following competing interests: [insert competing interests here]*<br><br>* typeset	
**Ethics Statement**<br><br>Enter an ethics statement for this submission. This statement is required if the study involved:<br><br>• Human participants<br>• Human specimens or tissue<br>• Vertebrate animals or cephalopods<br>• Vertebrate embryos or tissues<br>• Field research<br><br>Write "N/A" if the submission does not require an ethics statement.<br><br>General guidance is provided below. Consult the submission guidelines for detailed instructions. **Make sure that all information entered here is included in the Methods section of the manuscript.**	N/A

**Format for specific study types**<br><br>**Human Subject Research (involving human participants and/or tissue)**<br>• Give the name of the institutional review board or ethics committee that approved the study<br>• Include the approval number and/or a statement indicating approval of this research<br>• Indicate the form of consent obtained (written/oral) or the reason that consent was not obtained (e.g. the data were analyzed anonymously)<br><br>**Animal Research (involving vertebrate animals, embryos or tissues)**<br>• Provide the name of the Institutional Animal Care and Use Committee (IACUC) or other relevant ethics board that reviewed the study protocol, and indicate whether they approved this research or granted a formal waiver of ethical approval<br>• Include an approval number if one was obtained<br>• If the study involved *non-human primates*, add *additional details* about animal welfare and steps taken to ameliorate suffering<br>• If anesthesia, euthanasia, or any kind of animal sacrifice is part of the study, include briefly which substances and/or methods were applied<br><br>**Field Research**<br><br>Include the following details if this study involves the collection of plant, animal, or other materials from a natural setting:<br>• Field permit number<br>• Name of the institution or relevant body that granted permission	
**Data Availability**<br><br>Authors are required to make all data underlying the findings described fully available, without restriction, and from the time of publication. PLOS allows rare exceptions to address legal and ethical concerns. See the PLOS Data Policy and FAQ for detailed information.	Yes - all data are fully available without restriction

A Data Availability Statement describing where the data can be found is required at submission. Your answers to this question constitute the Data Availability Statement and **will be published in the article**, if accepted.<br><br>**Important:** Stating 'data available on request from the author' is not sufficient. If your data are only available upon request, select 'No' for the first question and explain your exceptional situation in the text box.<br><br>Do the authors confirm that all data underlying the findings described in their manuscript are fully available without restriction?	
**Describe where the data may be found in full sentences. If you are copying our sample text, replace any instances of XXX with the appropriate details.**<br><br>• If the data are **held or will be held in a public repository**, include URLs, accession numbers or DOIs. If this information will only be available after acceptance, indicate this by ticking the box below. For example: *All XXX files are available from the XXX database (accession number(s) XXX, XXX.*).<br>• If the data are all contained **within the manuscript and/or Supporting Information files**, enter the following: *All relevant data are within the manuscript and its Supporting Information files.*<br>• If neither of these applies but you are able to provide **details of access elsewhere**, with or without limitations, please do so. For example:<br><br>*Data cannot be shared publicly because of [XXX]. Data are available from the XXX Institutional Data Access / Ethics Committee (contact via XXX) for researchers who meet the criteria for access to confidential data.*<br><br>*The data underlying the results presented in the study are available from (include the name of the third party*	All relevant data are within the manuscript and its Supporting Information files.

and contact information or URL).<br>• This text is appropriate if the data are owned by a third party and authors do not have permission to share the data.<br><br>* typeset	
Additional data availability information:	Tick here if the URLs/accession numbers/DOIs will be available only after acceptance of the manuscript for publication so that we can ensure their inclusion before publication.

Powered by Editorial Manager® and ProduXion Manager® from Aries Systems Corporation

Manuscript Click here to access/download;Manuscript;manuscript210607.docx

RESEARCH ARTICLE

Transcriptome analysis of genes associated with flowering and fruit set in rabbiteye blueberry (*Vaccinium ashei*)

Xuan Gao[1¶], Lida Wang[1¶], Hong Zhang[1,2], Bo Zhu[1], Guosheng Lv[1], Jiaxin Xiao[1*]

1 Anhui Provincial Key Laboratory of the Conservation and Exploitation of Biological Resources, Anhui Normal University, WuHu, Anhui, China; 2 Anhui Microanaly Gene Limited Liability Company, Hefei, Anhui, China

*xjx0930@163.com (XJX)

¶ These authors contributed equally to this work.

Abstract

Flowering and fruit set are important traits affecting fruit quality and yield in rabbiteye blueberry (*Vaccinium ashei*). Intense efforts have been made to elucidate the influence of vernalization and phytohormones on flowering, but the molecular mechanisms of flowering and fruit set ~~from vegetative growth to reproductive development~~ remain unclear. We divided flowering and fruit set into leaf bud (S1), flower bud (S2), initial flower (S3), bloom flower (S4), pad fruit (S5), and cup fruit (S6) based on phenotype and identified 7, 1338, 69, 657, and 187 unique differentially expressed genes (DEGs) in comparisons of S2/S1,

S3/S2, S4/S3, S5/S4, and S6/S5, respectively. There were obviously more DEGs in S3/S2 and S5/S4 than in S2/S1, S4/S3, and S6/S5, suggesting that S3/S2 and S5/S4 represent major transitions from buds to fruit in blueberry. Gene ontology enrichment analysis indicated these DEGs were mostly enriched in phytohormone, transporter protein, and transcription factor categories. Gibberellic acid was important in early reproduction, while the expression of auxin response factors increased throughout the process from bud to fruit development. Transporter proteins were mainly associated with minerals in the early stage and sugars in later developmental stages. MYB86 expression was up-regulated in both flower development and initial fruit development, suggesting multiple functions in blueberry. Our study provides deep insights into the molecular mechanism underlying flowering and fruit set of rabbiteye blueberry.

Introduction

Flowering and fruit set are two of the most important events in the plant life cycle. The flowering process is a transition from vegetative growth to reproductive development, which directly affects ~~the formation of~~ fruit. ~~There are a lot of problems in blueberry production. Different sizes of fruit make the fruit low quality, while~~ falling flowers and fruits often lead to poor harvests. Understanding flowering and fruit set in blueberries will facilitate the improvement of fruit traits and yield.

Many previous studies have focused on the mechanism of flowering during plant development [1]. The transition from bud to flower, which plays a significant role in the plant life cycle, is overseen by a complicated regulatory network. The transition is controlled by both environmental cues and internal factors. Environment factors including light~~,~~ temperature~~, and drought~~ regulate the flowering process. For example, genes related to photoperiod such as *cryptochrome*, *EARLY FLOWERING (ELF)*, *LATE ELONGATED*

HYPOCOTYL (LHY), and *CONSTANS-LIKE (COL)* [2] are upregulated in apple during the flowering process. Genes related to vernalization and highly associated with the response to chilling are also upregulated at the initial stage of flowering, such as *VERNALIZATION INSENSITIVE (VIN)*, *SHORT VEGETATIVE PHASE (SVP)*, *FLOWERING TIME CONTROL PROTEIN FCA (FCA)*, and others [3]. *FLOWERING LOCUS T (FT)* plays a major role in promoting flowering. In a comparison of RNA-seq data from non-chilled, chilled, and late pink buds of southern highbush blueberry ~~(*Vaccinium ashei*)~~, *FT*, *Protein FD (FD)*, *TERMINAL FLOWER 1 (TFL1)*, *LEAFY (LFY)*, and MADS-box genes were identified as involved in chilling-mediated blueberry bud-break [3]. *FCA* and *FLOWERING TIME CONTROL PROTEIN (FPA)*, which are related to *FT*, also show high expression levels at the bud stage [2]. *FLOWERING LOCUS C (FLC)* and *FT* are regulated by the same pathway, while *FT* is repressed by *FLC* [4]. *FT* and *CYCLING DOF FACTOR1 (CDF1)* show high expression during early flowering [2]. *DELLA* and *MSII*, which are related to gibberellic acid (GA) regulation, also have high expression levels during the early stage of flowering [4]. Phytohormones such as GA, indole-3-acetic acid (IAA), and abscisic acid (ABA) represent another way to regulate the flowering process. Genes related to hormones show considerable changes in expression during the flowering process, such as *DELLA*, *GA2 oxidase* (*GA2OX*), *IAA3*, and genes encoding auxin transporters.

Some previous research has focused on fruit development [5]. *Vaccinium corymbosum* Caffeic acid *O*-methyltransferase (*VcCOMT*) family genes highly expressed during the development of blueberry fruit may contribute to greater fruit firmness [6]. Genes related to sugar and organic acids are upregulated during fruit development [7]. The

process of transition from flower to fruit involves the anthocyanin pathway, transcription factor-related genes, sugar-related genes, hormone-related genes, and photosynthesis. Flavonoids accumulate from the early fruit stage to the maturation stage [5]. High levels of expression of genes related to anthocyanin and carotene biosynthesis [8] may lead to pigment changes during fruit development. Some transcription factor-related genes such as MYB transcription factor (*MYB*) also have high expression levels at the fruit development stage. Glucose accumulates promptly during fruit development [5, 8]; however, the expression of *SUCROSE TRANSPORTER 2 (SUC2)* and *SWEET1,* which encode sugar transporters, is down-regulated [5]. Hormone pathways display complex regulation at this stage. Genes related to ethylene [5] and cytokinins [2, 8] are upregulated when plants transition from flower to fruit. On the contrary, genes related to IAA and auxin are down-regulated [2]. The regulation of GA is particularly complicated during the flowering process. Expression of *GA2OX* is upregulated [5], while expression of some other genes is down-regulated [2]. Photosynthesis may not matter during the transformation stage, as evidenced by many genes related to photosynthesis being expressed at lower levels from flowering to fruit set [2].

Many researchers have reported on changes in gene expression at the flower stage and fruit stage; however, few researchers have reported on the relationship between flowering and fruit set in blueberry. Gene expression patterns may be unique at different stages from bud to fruit. For example, *SVP* and *MAFs,* which are involved in vernalization, have a high expression level from the bud to the flower stage [4] but this decreases later. Blueberry yield depends not only on pollen performance but also on genes related to

hormones and on nutrition supplementation during the flower-fruit transition. In the present study, we generated RNA-seq data for six different stages of blueberry flower development from bud to fruit. We took advantage of techniques employed in Cufflinks using the genomic sequence of blueberry published in 2019 [9]. Our results may be more accurate than those from previous analyses employing Trinity. We aimed to determine the critical genes at each stage of blueberry development from bud to fruit, which will provide new insight into the mechanism of flowering and fruit set in blueberry and enable effective breeding of flowering and fruit set in blueberries.

Results

Identification and functional annotation of DEGs

We explored the expression levels of genes involved in blueberry flower development using Cuffdiff software. After filtering (false discovery rate <0.01), a total of 34 genes were differentially expressed, among which 7 were down-regulated and 27 were up-regulated in the S2 stage compared with the S1 stage (leaf bud to flower bud) (Figure 1B). A total of 2298 genes showed differential expression, with 1388 up-regulated and 910 down-regulated, in S3 compared with S2 (flower bud to initial flower). The comparison of S4 with S3 (initial flower to bloom flower) revealed 453 DEGs, with 180 up-regulated and 273 down-regulated. A total of 1764 genes displayed differential expression in S5 compared with S4 (bloom flower to pad fruit), with 784 up-regulated and 908 down-regulated. The comparison of S6 with S5 (pad fruit to cup fruit) identified 621 genes showing differential expression, with 323 up-regulated and 298 down-regulated. The number of DEGs in S3/S2 and S5/S4 was dramatically higher than that for the other three pairs.

The Venn diagram displays a comparative analysis of the DEGs described above (Figure 1A). We identified 5098 DEGs associated with transitions to different developmental stages from bud to fruit setting, with 7, 1338, 69, 657, and 187 DEGs being unique to S2/S1, S3/S2, S4/S3, S5/S4, S6/S5, respectively.

Figure 1. Analysis of differentially expressed genes (DEGs) between each library pair. (A) Venn diagram of the DEGs in comparisons of six samples (numbers in circles represent the numbers of DEGs in different stages). (B) Numbers of up-regulated (up) and down-regulated (down) genes in the comparisons of six samples. S1, S2, S3, S4, S5, and S6 represent leaf bud, flower bud, initial flower, bloom flower, pad fruit, and cup fruit, respectively.

Principal component analysis

Variability in DEGs between samples was studied using principal component analysis (PCA). Samples were grouped using data for all 48070 isoforms analyzed by Cufflinks. In general, replicate samples belonging to a given developmental stage clustered more closely together than samples belonging to different stages, except for leaf bud and flower bud (Figure 2). This result is consistent with the number of DEGs identified. As shown above, 2298 DEGs were identified between S2/S3 stages, which was dramatically higher than the number between other stages.

Figure 2. Principle component analysis (PCA) of variability in DEGs observed across samples. Colors are used to differentiate between samples: purple, blue, brown, red, green, and cyan represent leaf bud, flower bud, initial flower, bloom flower, pad fruit, and cup fruit, respectively.

GO analysis for different flower developmental stages

To determine the biological classification of DEGs, we performed functional and pathway enrichment analyses using ClusterProfiler R packages. GO analysis was performed by stage. Only seven unique genes were identified between stages S2 and S1 (from leaf bud to flower bud), which are listed in Table 1. Comparing S3 with S2, 8043 DEGs were significantly enriched in “aromatic compound catabolic process”, “cytoskeleton

Table 1. Differentially Expressed Genes between tages S1 and S2

ID	Description	Function	Regulation
VaccDscaff1294-processed-gene-0.1	RHO guanyl-nucleotide exchange factor 2	Acts downstream of PRK2 in the control of polarized pollen tube growth	up
VaccDscaff4-augustus-gene-144.39	Calcium-dependent phosphotriesterase superfamily protein	A novel plant protein required for pollen development, especially for proper exine formation	up
VaccDscaff47-augustus-gene-129.28	cinnamyl-alcohol dehydrogenase	Involved in lignin biosynthesis in the floral stem	up
VaccDscaff3-augustus-gene-10.22	LOB domain-containing protein 37	Transcription regulatory region sequence-specific DNA binding which involved in nitrogen-associated metabolism and growth.	up
VaccDscaff29-augustus-gene-121.23	ferritin	Important for iron homeostasis	up
VaccDscaff18-augustus-gene-236.20	Leucine-rich repeat protein kinase family protein	LRR-RLK is required for male fertility and the transduction in pollen tubes under high temperature.	down
VaccDscaff24-processed-gene-103.20	TCP family transcription factor	Plays a pivotal role in the control of morphogenesis of shoot organs, participates in ovule development.	down

organization”, and “vegetative to reproductive phase transition of meristem” in the Biological Process category (Figure 3A). The GO terms “vesicle” and “thylakoid” were prominent in the Cellular Component category, whereas “adenyl nucleotide binding” and

“active transmembrane transporter activity” were the apparent molecular functions of the DEGs. Comparing S4 with S3, “response to jasmonic acid”, “programmed cell death”, and “response to organonitrogen compounds” were notable under the Biology Process category (Figure 3B). In the comparison of S5 with S4, “mitotic cell cycle”, “cytoskeleton organization”, and “cell division” were dominant terms in the Biology Process category (Figure 3C). All these GO terms were related to cell growth, inferring that the number of cells began to rise at this stage. Abiotic and biotic stress related pathways also played an important role at the S5/S4 stage. Abiotic stress includes “response to water”, “response to light intensity”, and “response to heat” while biotic stress includes “response to virus”. The “flavonoid biosynthetic process” was another dominant term in pad fruit compared with bloom flower, indicating that flavonoids began to accumulate from the bloom stage to the early fruit stage. In the Cellular Component category, “intrinsic component of membrane” and “plant-type cell wall” were predominant terms. For the Molecular Function category, “ATP binding” and “adenyl nucleotide binding” were the most strikingly enriched terms. In the comparison of S6 with S5, “mitotic cell cycle”, “cytoskeleton organization”, and “cell division” were dominant terms in the Biology Process category, mostly similar to the bloom flower/pad fruit transition (Figure 3D). The difference between these comparisons was that stages S5 and S6 focused on organ development such as “regulation of post-embryonic development” and “regulation of shoot system development”.

Figure 3. GO analysis between developmental stages in blueberry. (A) S3/S2; (B) S4/S3; (C) S5/S4; (D) S6/S5. S2, S3, S4, S5, and S6 represent flower bud, initial flower, bloom flower, pad fruit, and cup fruit, respectively.

Candidate gene involved in the flowering process

We further conducted an overview hierarchical clustering analysis of the DEGs at each stage. DEGs in different replicates of the same stage exhibited similar expression patterns. However, comparisons of the same DEGs at different stages revealed differences in expression patterns (Figure 4). Seven unique DEGs were identified between stages S2 and S1 (leaf bud to flower bud), of which five genes were up-regulated and two genes were down-regulated (Table 1).

Figure 4. Gene expression profiles of candidate genes in four comparisons. (A) S3/S2; (B) S4/S3; (C) S5/S4; (D) S6/S5. S2, S3, S4, S5, and S6 represent flower bud, initial flower, bloom flower, pad fruit, and cup fruit, respectively. Green represents genes down-regulated and red represents genes up-regulated.

Genes related to pollen tube, floral stem, and iron transfer were up-regulated in flower bud compared with leaf bud, such as those encoding RHO guanyl-nucleotide exchange factor 2, calcium-dependent phosphotriesterase superfamily protein, and ferritin (Table 1). Genes related to male fertility and ovule development were down-regulated, such as those encoding leucine-rich repeat protein kinase family protein and TCP family transcription factor.

DEGs related to transporters, hormones, and photosynthesis showed considerable changes from bud stage to initial flower stage (S3/S2) (Figure 4A). Genes encoding many transporters, such as MATE (Multidrug and Toxic Compound Extrusion) efflux family protein, metal transporter, and sugar transporter, were expressed at higher levels in initial

flowers than in buds. On the contrary, the gene encoding a nitrate transporter was expressed at a lower level. DEGs related to auxin, jasmonic acid (JA), and ABA were up-regulated while genes related to ethylene and IAA were downregulated. DEGs related to photosynthesis such as AGL20 (AGAMOUS-like 20) and chlorophyll a-b binding were down-regulated.

DEGs related to hormones such as auxin, GA, and JA showed a sizeable change in expression from initial flowers to bloom flowers (S4/S3) (Figure 4B). The gene encoding the anther-specific protein LAT52, which is related to anther growth, was expressed at lower levels in bloom flowers. DEGs related to transcriptional factors such as zinc finger protein and BHLH70 (basic helix-loop-helix 70) were down-regulated as well.

DEGs related to anthocyanin, hormones, photosynthesis, and sugar transporters showed significant changes in expression from bloom flowers to pad fruit (S5/S4) (Figure 4C). Genes encoding anthocyanin regulatory protein and CHI (Chalcone isomerase) were expressed more highly in pad fruit than at the bloom stage. Genes related to cytokinins and auxin were up-regulated while genes related to ethylene were down-regulated. DEGs related to photosynthesis were expressed at higher levels in pad fruit than in bloom flowers. Transporters such as sugar transporters and ABC transporters were all down-regulated in pad fruit compared with bloom flowers.

Expression levels of DEGs related to pathogenesis, hormones, photosynthesis, sugars, transcriptional factors, and flower growth were changed significantly in cup fruit (S6) compared with pad fruit (S5) (Figure 4D). Disease-resistance proteins and pathogenesis-related protein were down-regulated in cup fruit compared with pad fruit.

DEGs related to GA and JA were also down-regulated, while genes associated with auxin were mainly up-regulated throughout the whole process from bud to fruit. Transporter genes such as those encoding MYB and zinc finger/C3HC4-type family proteins were up-regulated while those encoding BHLH proteins were down-regulated. DEGs related to flower growth such as those encoding Anther-specific protein, AGL 31 (AGAMOUS-like 31), and COL 9 (CONSTANS-like 9) were all down-regulated.

Validation of DEGs by quantitative reverse-transcription PCR (qRT-PCR)

To confirm the accuracy of the RNA-seq results, we examined the transcription of nine putative genes involved in flower development through a supporting qRT-PCR experiment (Figure 5). Transcriptional levels of AUX2 (auxin transporter-like protein 2) and CHI (Chalcone-flavanone isomerase family protein) were significantly up-regulated ($p < 0.05$) during the process of flowering and fruit set compared with earlier developmental stages, in agreement with the alterations in gene expression detected by the transcriptome analysis. The transcriptional level of CAB (encoding Chlorophyll A-B binding family protein) decreased from the bud stage to pad fruit and then increased slightly again in cup fruit. The transcriptional level of chlorophyllase I peaked at the pad fruit stage and then decreased. These nine genes provided more evidence to support the reliability of our RNA sequencing data.

Figure 5. Validation of gene expression by qRT-PCR. Expression levels of nine candidate genes—*ABA3*, *AUX2* (*auxin transporter-like protein 2*), *CAB* (*chlorophyll A-B binding family protein*), *CHI*

(*chalcone-flavanone isomerase family protein*), *chlorophyllase I*, *Disease resistance protein* (CC-NBS-LRR class) family member, *IAA11*, *MATE* (encoding a MATE efflux family protein), and *SUT4* (*sucrose transporter 4*)—were determined at each stage using qRT-PCR analyses. The bars and error bars represent the qPCR while the line represents the results of RNA-seq.

Discussion

Uncovering the rules for flowering and fruit set in blueberry will facilitate improvement of fruit traits. The rapid advances and reduction in cost of sequencing offer exclusive opportunities for exploring the molecular mechanisms underlying flowering and fruit set processes in blueberry species. Furthermore, recent publication of the genomic sequence of blueberry increases the accuracy of transcriptome analysis [10]. Previous studies have only focused on two [11] or three [3] stages during the development of flowering in blueberry. The lack of comparative transcriptome analysis between different stages of flowering and fruit set in blueberry impedes further functional genomics and molecular biology research. In this study, we divided flowering and fruit set into six phases distinguished by the morphology of flowers, including bud stages, flower stages, and fruit stages.

Comparing the number of DEGs at different stages, we found significantly more genes regulated from bud to flower and from flower to fruit than during other stage transitions (Figure 1A). The genes differentially expressed during these two stage transitions may play important roles in reproductive growth in blueberry.

DEGs were remarkably enriched in the “aromatic compound catabolic process” from bud to initial flower stages, indicating that the production of aroma compounds was

increased in preparation for flowering (Figure 3A). ABA can enhance the expression of betaine aldehyde dehydrogenase 2 (BADH2), which is related to flower fragrance in most plants [12]. In this study, ABA3, which acts as a key regulator of ABA biosynthesis [13], was upregulated in initial flowers of blueberry (Figure 4A). We speculate that aromatic compounds were increased via regulating the content of ABA. Some transfer genes were upregulated at this developmental stage, such as those encoding the MATE efflux family protein, high affinity K+ transporter 5, and heavy metal transport/detoxification superfamily protein, indicating that blueberry requires mineral elements during the transition from buds to initial flowers. Expression of genes encoding sugar transporter proteins was also up-regulated, while that of genes encoding nitrate transporters was down-regulated, suggesting that an increase in C / N ratio is beneficial for flower bud formation and flowering. This is consistent with findings in orchid, where C/N ratio plays an important role in shaping flower structure [14]. Hormone-related genes have complicated roles throughout the flowering and fruit set period. Hormone genes related to GA, auxin, JA, and ABA were up-regulated while genes related to ethylene and IAA were down-regulated from the bud to initial flower stage. The gene encoding Gibberellin-regulated protein 1 (GAST1) protein [15], which may be involved in seed germination, flowering, and seed maturation was up-regulated in our study. Genes encoding SAUR-like auxin-responsive protein and auxin transporter-like protein were up-regulated, indicating that auxin may play an important role from in the transition from bud to initial flower stage. IAA11 proteins act as repressors of early auxin response genes [16]. Down-regulation of IAA11 and up-regulated of genes encoding auxin transporter proteins suggests that the auxin content increased during this

stage. Auxin response genes were up-regulated, consistent with previous reports [17], showing the importance of auxin at the initial flower stage. Photosynthesis-related genes such as chlorophyll a-b binding family protein were downregulated, consistent with previous reports [2], indicating that photosynthesis may have little effect on the transition from buds to initial flowering. Consistent overexpression of FT protein may impede further development of blueberry flowers [18]. AGAMOUS-like 20 (AGL20), involved in the FT-regulated pathway, may integrate signals from the photoperiod, vernalization, and autonomous floral induction pathways [19]. In our study, we identified no significant difference in expression of genes related to FT, except AGL20, from the bud to initial flower stages. Expression of AGL20 decreased, suggesting that the flowering process regulated by FT is just before the initial flower stage or even earlier. After all, overexpression of FT may lead to abnormal flowering in blueberry [20].

The transition from flowering to fruit set is another important transition in blueberry. Genes encoding Anthocyanin regulatory C1 protein-like and CHI (chalcone-flavanone isomerase family protein) were upregulated from bloom flower to pad fruit stages (Figure 4C and Figure 5). These two genes are both involved in the flavone pathway, which may increase the content of anthocyanin. Up-regulation of genes encoding cytokinin response factor 4, auxin response factor 18, and auxin efflux carrier family protein suggests that cytokinin and auxin may play important roles at the pad fruit stage (Figure 4C). Down-regulation of ethylene-responsive transcription factors at this stage may reduce the content of ethylene to avoid early fruit falling. Photosynthesis increases from initial flower stage to pad fruit stage, suggesting that supplementation of carbohydrate accumulation is required

for growth of young fruit. Genes encoding transporters such as sucrose-proton symporter 2 and sugar transporter 14 were down-regulated in pad fruit compared with initial flowers, inferring that sugar may accumulate in the fruit tissue. The expression of hormone genes related to auxin was increased during both the bud to flower transition and the flower to fruit transition (Figure 4A and Figure 4C). On the contrary, expression of genes encoding transporters for sugar was increased at the bud to flower transition but decreased at the flower to fruit transition. This may indicate that auxin plays an important role in enhancing fruit growth in blueberry.

We observed few changes in gene expression from leaf bud to flower bud in blueberry. Genes related to pollen development were up-regulated during this transition, indicating that the leaf bud was preparing for flowering (Table 1).

From the pad fruit to the cup fruit stage, the fruit expands quickly and then remains in the cup stage for a long time. Genes encoding some pathogenesis-related proteins such as disease resistance protein and pathogenesis-related protein 5 were down-regulated during this transition, indicating that blueberry is vulnerable to disease at the cup fruit stage (Figure 4D). Expression of auxin-related genes such as SAUR-like auxin-responsive protein family was increased, with genes related to GA and JA down-regulated. Some transcriptional factors were regulated in a complex way. Genes encoding MYB86 and Zinc finger/C3HC4-type family protein were up-regulated while BHLH was down-regulated at the cup fruit stage compared with pad fruit. NAC031 is involved in the molecular mechanisms regulating shoot apical meristem (SAM) formation and acts as an inhibitor of cell division [21]. AGAMOUS-like 31 (AGL 31) acts as a floral repressor [22]. Flowering

time control protein FCA regulates flowering time, seed size, and cell volume. CONSTANS-like 9 (COL9) may be involved in the light input to the circadian clock [23]. All of these genes were down-regulated during the pad fruit to the cup fruit transition, indicating that the flowering process has just completed at this stage.

More interestingly, MYB86 gene expression was up-regulated during both flower development and initial fruit development (Figure 4B andFigure 4C). MYB86 expression was found to be concurrent with anthocyanin accumulation in fruits of Fragaria vesca [24]. MYB86 is also involved in secondary cell wall biosynthesis in Populus [25]. Therefore, MYB86 may have multiple functions in plant development benefitting flower formation and anthocyanin accumulation in blueberry. However, this needs further research to confirm.

In this study, we demonstrated that hormones, anthocyanin, and pathogenesis are involved in the blueberry flowering and fruit set process. Hormones may have a complex function because expression of genes encoding hormones changed frequently at different developmental stages. For example, Expression of GA-related genes peaked at the early stage of flower development, but decreased later. Expression of IAA-related genes went up and down during the development of flowering. However, expression of AUX2 increased steady through the whole flower and fruit set process in blueberry. This was identified thr0ugh both RNA-seq data and qRT-PCR, suggesting that AUX2 plays an important role in the flowering process. Expression of genes related to anthocyanin was increased during later stages of blueberry flowering while that of pathogenesis-related genes was decreased, which may suggest that anthocyanin makes up some deficiency caused by lack of pathogen resistance. However, this needs further research.

Materials and Methods

Blueberry materials

Nine-year-old shrubs of rabbiteye ‘Brightwell’ blueberry (*Vaccinium ashei*) were cultivated in Nanling County, Anhui Province, China. Flowering and fruit set were divided into six stages based on morphological characterization (Figure 6). Leaf bud (S1) and flower bud (S2) were collected in early and late March, respectively. Initial flower (S3) and bloom flower (S4) were collected in April when the blueberry was flowering. Pad fruit (S5) was collected in late April when fruit began to form and looked similar to a pad. Cup fruit (S6) was collected in early May and represented a stage present for a long time.

Figure 6. Samples of *Vaccinium ashei* for RNA-seq. S1. leaf bud; S2. flower bud; S3. initial flower; S4. bloom flower; S5. pad fruit; S6. cup fruit.

Construction of cDNA library and Illumina Sequencing

Total RNA was extracted using Trizol reagent (Invitrogen), and genomic DNA contamination was removed using DNase I (TaKaRa, Dalian, China). RNA quality was verified using agarose gel electrophoresis and a Bioanalyzer 2100 (Aglient Technologies, Palo Alto, CA, USA). For cDNA library construction, mRNA was enriched using oligo (dT) magnetic beads and then broken into smaller pieces using fragmentation buffer (GeneChip® WT Terminal labeling kit; Affymetrix; P/N 900671). cDNA libraries were sequenced using an Illumina HiSeq2000 platform at WeiFen GENE Co., ChaoHu, China. The reference genome and gene model annotation files were downloaded directly from the

blueberry genome website (https://www.vaccinium.org/crop/blueberry). An index of the reference genome was built using Bowtie software (v2.2.8). Raw reads were filtered to obtain high-quality reads by removing low-quality reads (Q value <20). The high-quality reads were mapped onto the highbush blueberry (*Vaccinium corymbosum*) reference genome using TopHat [26]. Assembled transcripts were annotated using the Cuffcompare program from the Cufflinks package. Gene expression was quantified in terms of reads per kilobase of exon model per million mapped reads (RPKM) values. Cuffdiff (v2.1.1) was used to calculate RPKM of mRNAs. The R package cummeRbund was used to count the number of reads mapped to each gene. The RPKM value for each gene was then calculated based on the length of the gene and the number of reads mapped to it. Transcripts with $p<0.05$ were considered to be significantly differentially expressed.

GO analysis and identification of DEGs

A gene ontology (GO) functional analysis was performed to identify the biological processes most strongly represented by the DEGs. GO enrichment analysis of DEGs was performed using the ClusterProlifer package in R software [27].

Validation of RNA-seq data by quantitative reverse-transcription PCR

The reliability of DEGs/transcripts identified through RNA sequencing was evaluated through quantitative RT-PCR analysis of nine selected transcripts. Reverse transcription of RNA to cDNA was performed using SuperScript II reverse transcriptase (Invitrogen). The resulting cDNA from 1 μg of RNA was diluted in water, and 1 μl of sample (25 ng) was used

for each PCR. Three replicated RNA samples from the flower tissues of each blueberry stage were used. Primers were designed using Primer premier v5.0 software (Applied Biosystems, Foster City, CA, USA). Quantitative RT-PCR was performed in triplicate using the SYBR Green System (Thermo Fisher). Each 25 μl reaction mixture contained 25 ng cDNA, 100 nM primers, and 12.5 μl of 2 X SYBR Green master mix. Reaction conditions for all primer pairs were 95 °C for 10 min, 40 cycles of 30 s at 95 °C, 60 s at 60 °C, and 60 s at 72 °C, followed by 1 cycle of 60 s at 95 °C, 30 s at 55 °C, and 30 s at 95 °C. The specificity of the reaction for each primer pair was determined using a melting curve. Relative expression was normalized using the eukaryotic translation initiation factor 3 subunit H and was calculated using the $2^{-\Delta\Delta Ct}$ method.

Conclusions

Hormones play an essential role in regulating flowering and early fruit development during the blueberry flowering and fruit set process. Expression of GA-related genes was upregulated at the initial flower stage but decreased at later stages, suggesting that regulation of GA may only be effective during the early stage of the flowering process. Expression of auxin-related genes increased throughout the process from bud to fruit, suggesting that auxin plays a very important role in the flowering process. Of particular interest, expression of *FT*, an inducer of flowering, showed no significant change during the bud to fruit stage. *AGL20,* which is regulated by *FT*, was down-regulated from the bud to initial flower stage, suggesting that *FT* may only affect the flowering process at the early stage, similar to GA. Anthocyanin accumulation may begin at the cup fruit stage because anthocyanin biosynthesis was up-regulated at this stage. Flowering-development-related

genes were constitutively expressed after the flower bloom, inferring that the flowering process is not finished until the growth of the pad fruit. Genes encoding pathogenesis-related proteins were down-regulated at the cup fruit stage, suggesting that this blueberry may be susceptible to disease.

References

1. Chen X, Mao X, Huang P, Fang S. Morphological Characterization of Flower Buds Development and Related Gene Expression Profiling at Bud Break Stage in Heterodichogamous Cyclocarya paliurus (Batal.) Iljinskaja. Genes (Basel). 2019;10(10). Epub 2019/10/20. doi: 10.3390/genes10100818. PubMed PMID: 31627470; PubMed Central PMCID: PMCPMC6827045.

2. Liu K, Feng S, Pan Y, Zhong J, Chen Y, Yuan C, et al. Transcriptome Analysis and Identification of Genes Associated with Floral Transition and Flower Development in Sugar Apple (Annona squamosa L.). Front Plant Sci. 2016;7:1695. Epub 2016/11/25. doi: 10.3389/fpls.2016.01695. PubMed PMID: 27881993; PubMed Central PMCID: PMC5101194.

3. Song GQ, Chen Q. Comparative transcriptome analysis of nonchilled, chilled, and late-pink bud reveals flowering pathway genes involved in chilling-mediated flowering in blueberry. BMC Plant Biol. 2018;18(1):98. Epub 2018/06/02. doi: 10.1186/s12870-018-1311-8 10.1186/s12870-018-1311-8 [pii]. PubMed PMID: 29855262; PubMed Central PMCID: PMC5984463.

4. Wang S, Li Z, Jin W, Fang Y, Yang Q, Xiang J. Transcriptome analysis and identification of genes associated with flower development in Rhododendron pulchrum Sweet (Ericaceae). Gene. 2018;679:108-18. Epub 2018/09/04. doi: S0378-1119(18)30952-1 [pii] 10.1016/j.gene.2018.08.083. PubMed PMID: 30176315.

5. Domingos S, Fino J, Paulo OS, Oliveira CM, Goulao LF. Molecular candidates for early-stage flower-to-fruit transition in stenospermocarpic table grape (Vitis vinifera L.) inflorescences ascribed by differential transcriptome and metabolome profiles. Plant Sci. 2016;244:40-56. Epub 2016/01/27. doi: S0168-9452(15)30116-3 [pii] 10.1016/j.plantsci.2015.12.009. PubMed PMID: 26810452.

6. Liu Y, Wang Y, Pei J, Li Y, Sun H. Genome-wide identification and characterization of COMT gene family during the development of blueberry fruit. BMC Plant Biol. 2021;21(1):5. Epub 2021/01/08. doi: 10.1186/s12870-020-02767-9. PubMed PMID: 33407129; PubMed Central PMCID: PMCPMC7789564.

7. Li X, Li C, Sun J, Jackson A. Dynamic changes of enzymes involved in sugar and organic acid level modification during blueberry fruit maturation. Food Chem. 2020;309:125617. Epub 2019/11/14. doi: 10.1016/j.foodchem.2019.125617. PubMed PMID: 31718833.

8. Singh VK, Garg R, Jain M. A global view of transcriptome dynamics during flower development in chickpea by deep sequencing. Plant Biotechnol J. 2013;11(6):691-701. Epub 2013/04/05. doi: 10.1111/pbi.12059. PubMed PMID: 23551980.

9. Colle M, Leisner CP, Wai CM, Ou S, Bird KA, Wang J, et al. Haplotype-phased genome and evolution of phytonutrient pathways of tetraploid blueberry. Gigascience. 2019;8(3). Epub

2019/02/05. doi: 10.1093/gigascience/giz012. PubMed PMID: 30715294; PubMed Central PMCID: PMCPMC6423372.

10. Colle M, Leisner CP, Wai CM, Ou S, Bird KA, Wang J, et al. Haplotype-phased genome and evolution of phytonutrient pathways of tetraploid blueberry. Gigascience. 2019;8(3). doi: 10.1093/gigascience/giz012.

11. Silva S, Costa EM, Coelho MC, Morais RM, Pintado ME. Variation of anthocyanins and other major phenolic compounds throughout the ripening of four Portuguese blueberry (Vaccinium corymbosum L) cultivars. Nat Prod Res. 2017;31(1):93-8. Epub 2016/10/01. doi: 10.1080/14786419.2016.1209668. PubMed PMID: 27686738.

12. Khandagale KS, Chavhan R, Nadaf AB. RNAi-mediated down regulation of BADH2 gene for expression of 2-acetyl-1-pyrroline in non-scented indica rice IR-64 (Oryza sativa L.). 3 Biotech. 2020;10(4):145. Epub 2020/03/18. doi: 10.1007/s13205-020-2131-8. PubMed PMID: 32181107; PubMed Central PMCID: PMCPMC7052105.

13. Bittner F, Oreb M, Mendel RR. ABA3 is a molybdenum cofactor sulfurase required for activation of aldehyde oxidase and xanthine dehydrogenase in Arabidopsis thaliana. J Biol Chem. 2001;276(44):40381-4. Epub 2001/09/13. doi: 10.1074/jbc.C100472200. PubMed PMID: 11553608.

14. Brzosko E, Bajguz A, Chmur M, Burzynska J, Jermakowicz E, Mirski P, et al. How Are the Flower Structure and Nectar Composition of the Generalistic Orchid Neottia ovata Adapted to a Wide Range of Pollinators? Int J Mol Sci. 2021;22(4). Epub 2021/03/07. doi: 10.3390/ijms22042214. PubMed PMID: 33672302; PubMed Central PMCID: PMCPMC7926835.

15. Herzog M, Dorne AM, Grellet F. GASA, a gibberellin-regulated gene family from Arabidopsis thaliana related to the tomato GAST1 gene. Plant Mol Biol. 1995;27(4):743-52. Epub 1995/02/01. doi: 10.1007/BF00020227. PubMed PMID: 7727751.

16. Liscum E, Reed JW. Genetics of Aux/IAA and ARF action in plant growth and development. Plant Mol Biol. 2002;49(3-4):387-400. Epub 2002/05/31. PubMed PMID: 12036262.

17. Sun R, Wang S, Ma D, Li Y, Liu C. Genome-Wide Analysis of Cotton Auxin Early Response Gene Families and Their Roles in Somatic Embryogenesis. Genes (Basel). 2019;10(10). Epub 2019/09/25. doi: 10.3390/genes10100730. PubMed PMID: 31547015; PubMed Central PMCID: PMCPMC6827057.

18. Walworth AE, Chai B, Song GQ. Transcript Profile of Flowering Regulatory Genes in VcFT-Overexpressing Blueberry Plants. PLoS One. 2016;11(6):e0156993. Epub 2016/06/09. doi: 10.1371/journal.pone.0156993

PONE-D-16-09377 [pii]. PubMed PMID: 27271296; PubMed Central PMCID: PMC4896415.

19. Lee H, Suh SS, Park E, Cho E, Ahn JH, Kim SG, et al. The AGAMOUS-LIKE 20 MADS domain protein integrates floral inductive pathways in Arabidopsis. Genes Dev. 2000;14(18):2366-76. Epub 2000/09/20. doi: 10.1101/gad.813600. PubMed PMID: 10995392; PubMed Central PMCID: PMCPMC316936.

20. Gao X, Walworth AE, Mackie C, Song GQ. Overexpression of blueberry FLOWERING LOCUS T is associated with changes in the expression of phytohormone-related genes in blueberry plants. Hortic Res. 2016;3:16053. Epub 2016/11/08. doi: 10.1038/hortres.2016.53. PubMed PMID: 27818778; PubMed Central PMCID: PMCPMC5080838.

21. Fang ZZ, Zhou DR, Ye XF, Jiang CC, Pan SL. Identification of Candidate Anthocyanin-Related Genes by Transcriptomic Analysis of 'Furongli' Plum (Prunus salicina Lindl.) during Fruit Ripening Using RNA-Seq. Front Plant Sci. 2016;7:1338. Epub 2016/09/16. doi: 10.3389/fpls.2016.01338. PubMed PMID: 27630660; PubMed Central PMCID: PMC5005409.

22. Rosloski SM, Jali SS, Balasubramanian S, Weigel D, Grbic V. Natural diversity in flowering responses of Arabidopsis thaliana caused by variation in a tandem gene array. Genetics. 2010;186(1):263-76. Epub 2010/06/17. doi: 10.1534/genetics.110.116392. PubMed PMID: 20551443; PubMed Central PMCID: PMCPMC2940291.

23. Li G, Siddiqui H, Teng Y, Lin R, Wan XY, Li J, et al. Coordinated transcriptional regulation underlying the circadian clock in Arabidopsis. Nat Cell Biol. 2011;13(5):616-22. Epub 2011/04/19. doi: 10.1038/ncb2219. PubMed PMID: 21499259.

24. Zhang Y, Li W, Dou Y, Zhang J, Jiang G, Miao L, et al. Transcript Quantification by RNA-Seq Reveals Differentially Expressed Genes in the Red and Yellow Fruits of Fragaria vesca. PLoS One. 2015;10(12):e0144356. Epub 2015/12/05. doi: 10.1371/journal.pone.0144356. PubMed PMID: 26636322; PubMed Central PMCID: PMCPMC4670188.

25. Wessels B, Seyfferth C, Escamez S, Vain T, Antos K, Vahala J, et al. An AP2/ERF transcription factor ERF139 coordinates xylem cell expansion and secondary cell wall deposition. New Phytol. 2019;224(4):1585-99. Epub 2019/05/28. doi: 10.1111/nph.15960. PubMed PMID: 31125440.

26. Trapnell C, Roberts A, Goff L, Pertea G, Kim D, Kelley DR, et al. Differential gene and transcript expression analysis of RNA-seq experiments with TopHat and Cufflinks. Nat Protoc. 2012;7(3):562-78. Epub 2012/03/03. doi: nprot.2012.016 [pii]

10.1038/nprot.2012.016. PubMed PMID: 22383036; PubMed Central PMCID: PMC3334321.

27. Yu G, Wang LG, Han Y, He QY. clusterProfiler: an R package for comparing biological themes among gene clusters. OMICS. 2012;16(5):284-7. Epub 2012/03/30. doi: 10.1089/omi.2011.0118. PubMed PMID: 22455463; PubMed Central PMCID: PMCPMC3339379.

Click here to access/download;Figure;Fig 1.tif

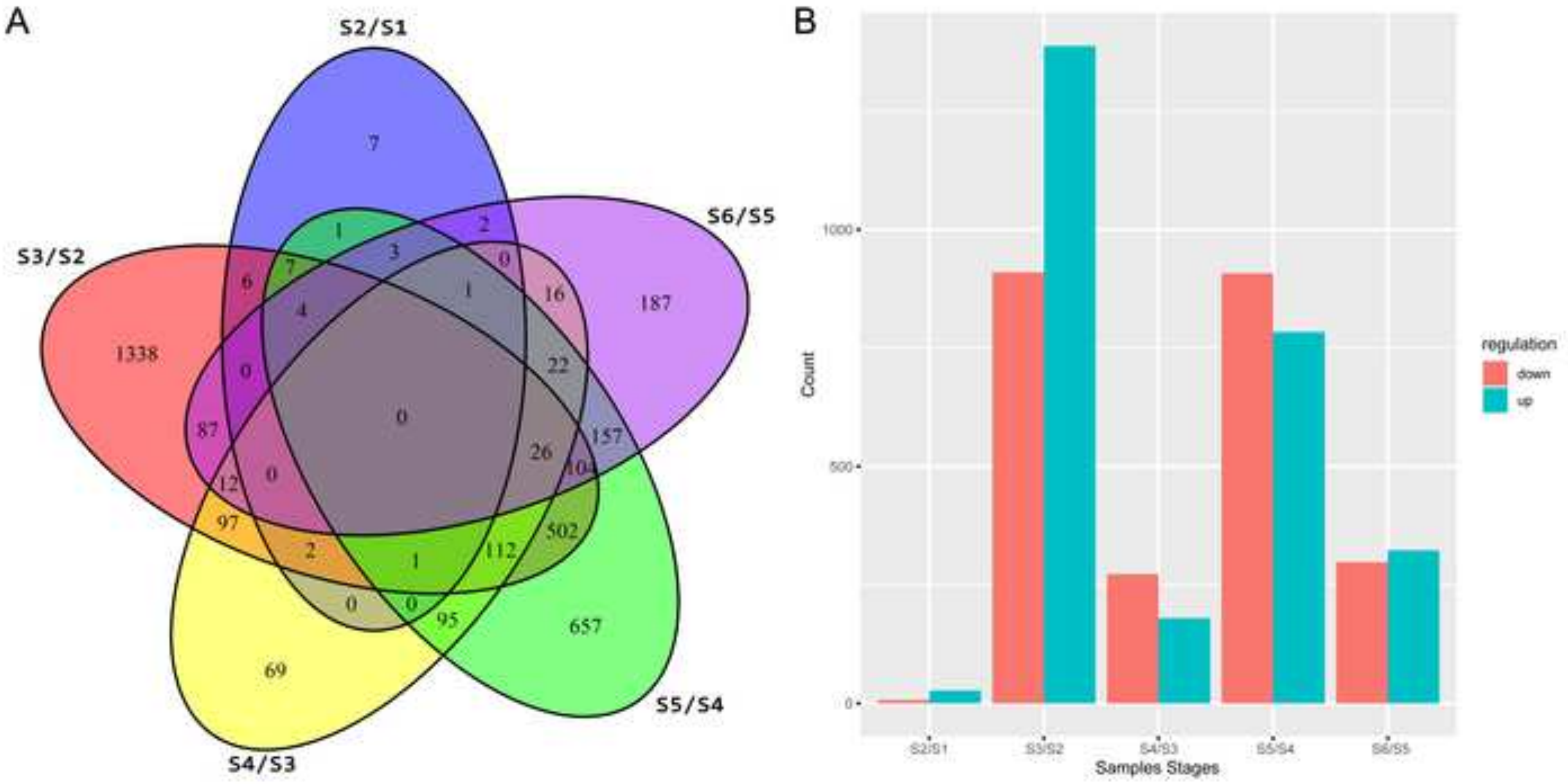

Figure 2

Click here to access/download;Figure;Fig 2.tif

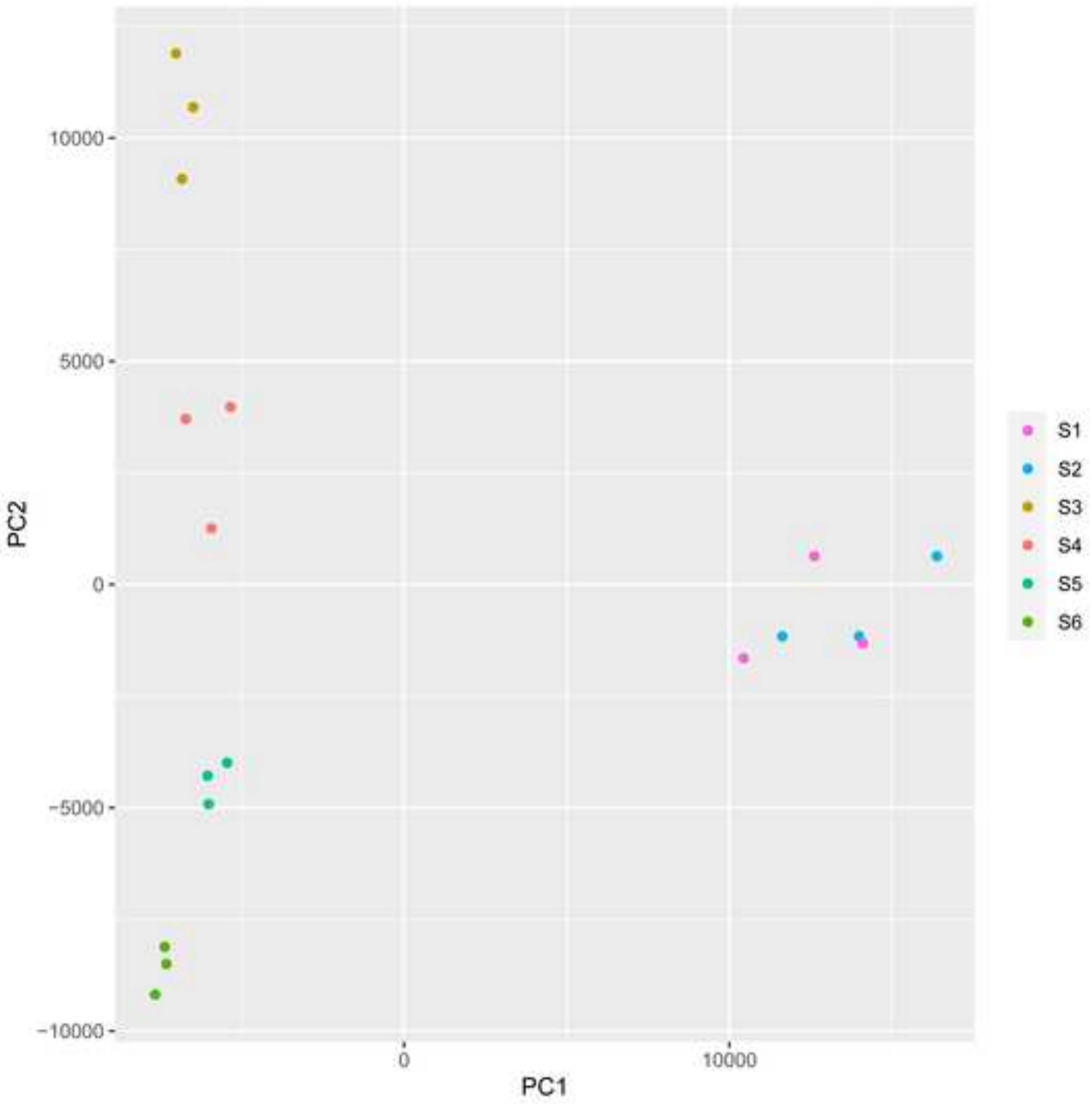

Figure 3

Click here to access/download;Figure;Fig 3.tif

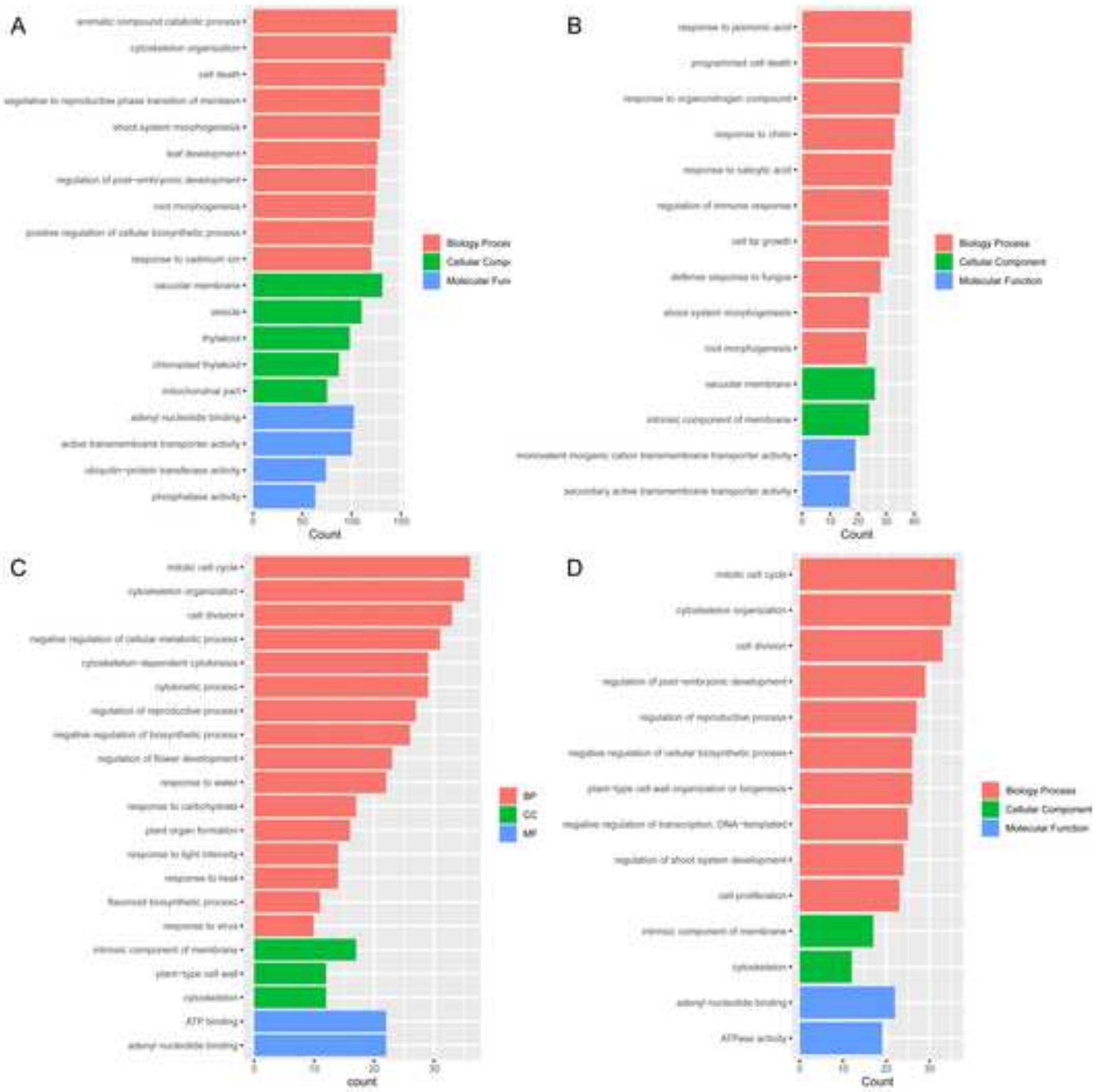

Figure 4

Click here to access/download;Figure;Fig 4.tif

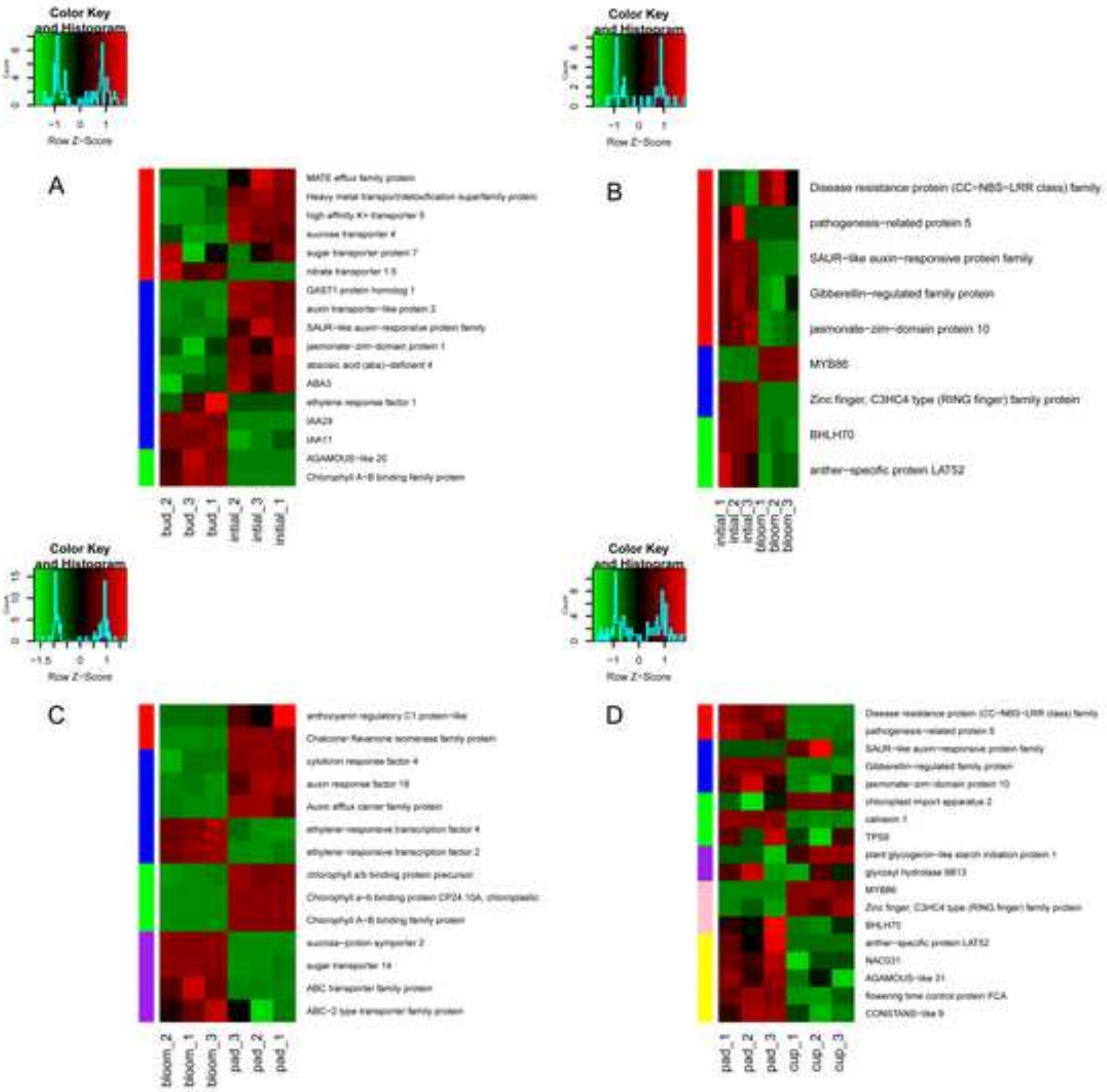

Figure 5

Click here to access/download;Figure;Fig 5.tif

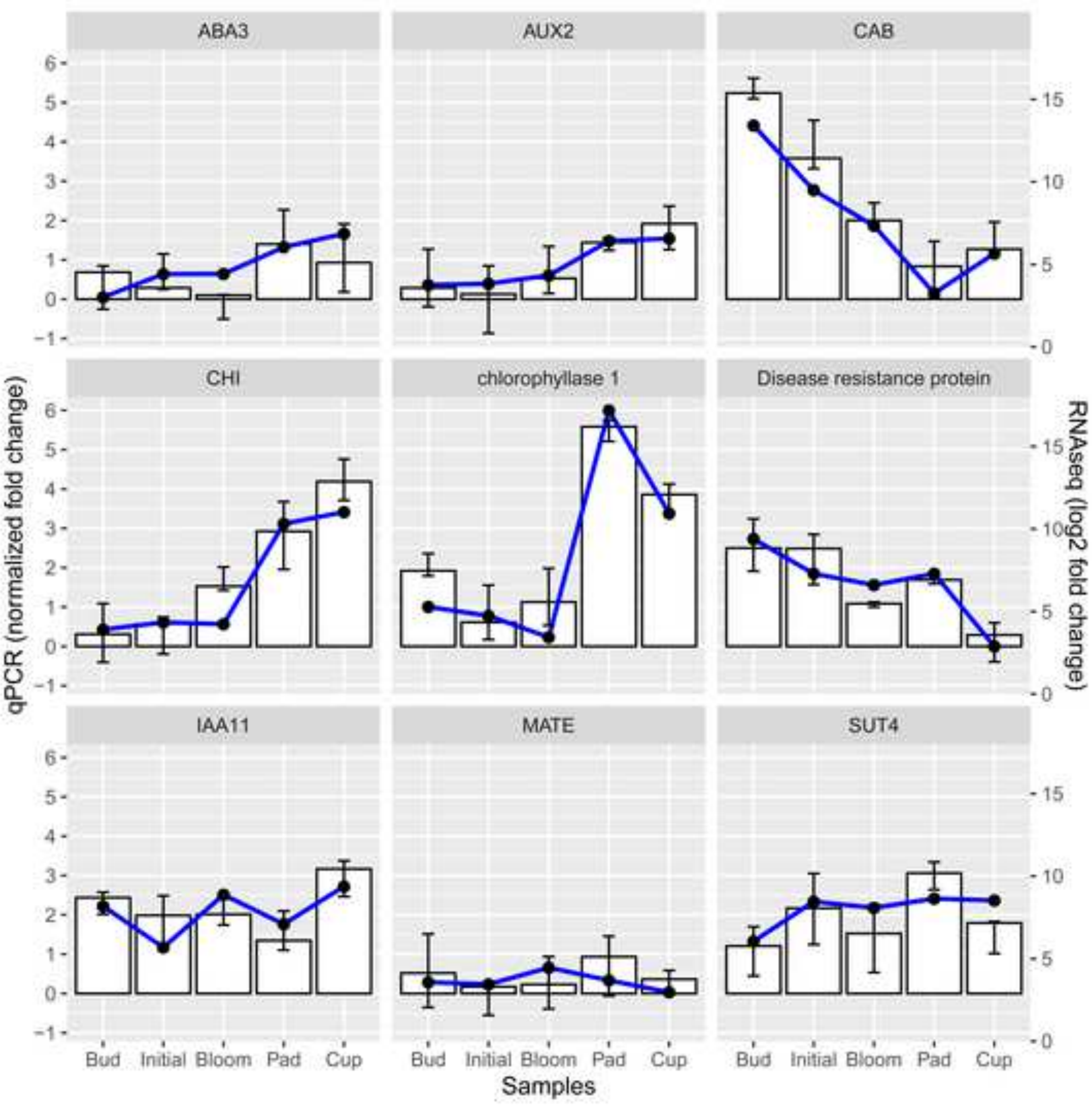

Figure 6

Click here to access/download;Figure;Fig 6.tif

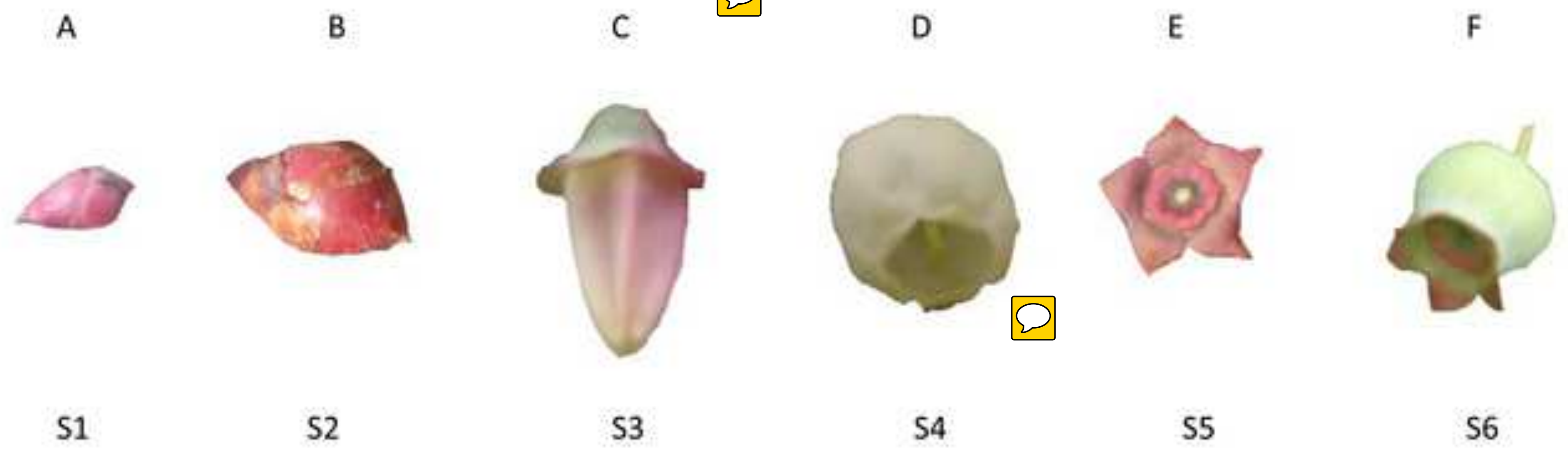

Supplement: Attachment — Submitted filename: PONE-D-21-18707_reviewer1.pdf [file pone.0259119.s012.pdf]
