# Supplementary material for: Transcriptome analysis and identification of genes associated with floral transition and fruit development in rabbiteye blueberry (Vaccinium ashei)
Source: PLoS One. 2021 Oct 28;16(10):e0259119. doi: 10.1371/journal.pone.0259119 (PMC8553168; doi:10.1371/journal.pone.0259119)
Supplement: Attachment — Submitted filename: Response to Reviewers.docx [file pone.0259119.s013.docx]

Dear Prof. Sussmilch

Re: Manuscript reference PONE-D-21-18707

Please find attached a revised version of our manuscript “Transcriptome analysis and identification of genes associated with floral transition and fruit development in rabbiteye blueberry (Vaccinium ashei)”, which we would like to resubmit for publication as a research paper in *PLOS ONE.*

Your comments, as well as those of the communicating editor and reviewers, were highly insightful and enabled us to greatly improve the quality of our manuscript. In the following pages, we provide a point-by-point response to all the comments we received.

We have extensively revised the original manuscript, and the changes can be found in red color font in the text. We hope that the revisions in the manuscript and our accompanying responses will be sufficient to make our manuscript suitable for publication in *PLOS ONE.*

We look forward to hearing from you at your earliest convenience.

Yours sincerely,

Xuan Gao, Prof. Dr.

College of Life Sciences, Anhu Normal University

Wuhu, Anhui Province, China

E-mail: gaoxuan@ahnu.edu.cn

1. Please ensure that your manuscript meets PLOS ONE's style requirements, including those for file naming. The PLOS ONE style templates can be found at

<https://journals.plos.org/plosone/s/file?id=wjVg/PLOSOne_formatting_sample_main_body.pdf>

and

<https://journals.plos.org/plosone/s/file?id=ba62/PLOSOne_formatting_sample_title_authors_affiliations.pdf>

**Response: Thank you for your suggestion. We have adjusted the format of the manuscript**

**including those for file naming according to your suggestion.**

2. Thank you for stating the following financial disclosure:

"The funders had no role in study design, data collection and analysis, decision to publish, or preparation of the manuscript."

At this time, please address the following queries:

a) Please clarify the sources of funding (financial or material support) for your study. List the grants or organizations that supported your study, including funding received from your institution.

b) State what role the funders took in the study. If the funders had no role in your study, please state: “The funders had no role in study design, data collection and analysis, decision to publish, or preparation of the manuscript.”

c) If any authors received a salary from any of your funders, please state which authors and which funders.

d) If you did not receive any funding for this study, please state: “The authors received no specific funding for this work.”

Please include your amended statements within your cover letter; we will change the online submission form on your behalf.

**Response: In response to your suggestion, we have added the financial disclosure: “This work was supported by the National Natural Science Foundation of China (31101148) and the foundation of Anhui Normal University (2010cxjj14)”** **within our cover letter.**

3. In your Data Availability statement, you have not specified where the minimal data set underlying the results described in your manuscript can be found. PLOS defines a study's minimal data set as the underlying data used to reach the conclusions drawn in the manuscript and any additional data required to replicate the reported study findings in their entirety. All PLOS journals require that the minimal data set be made fully available. For more information about our data policy, please see http://journals.plos.org/plosone/s/data-availability.

"Upon re-submitting your revised manuscript, please upload your study’s minimal underlying data set as either Supporting Information files or to a stable, public repository and include the relevant URLs, DOIs, or accession numbers within your revised cover letter. For a list of acceptable repositories, please see http://journals.plos.org/plosone/s/data-availability#loc-recommended-repositories. Any potentially identifying patient information must be fully anonymized.

Important: If there are ethical or legal restrictions to sharing your data publicly, please explain these restrictions in detail. Please see our guidelines for more information on what we consider unacceptable restrictions to publicly sharing data: http://journals.plos.org/plosone/s/data-availability#loc-unacceptable-data-access-restrictions. Note that it is not acceptable for the authors to be the sole named individuals responsible for ensuring data access.

We will update your Data Availability statement to reflect the information you provide in your cover letter.

4. We note that you have stated that you will provide repository information for your data at acceptance. Should your manuscript be accepted for publication, we will hold it until you provide the relevant accession numbers or DOIs necessary to access your data. If you wish to make changes to your Data Availability statement, please describe these changes in your cover letter and we will update your Data Availability statement to reflect the information you provide.

**Response: In response to questions 3-4, we have added the following data availability statement: “The raw reads data are available in the National Center for Biotechnology Information NCBI sequence read archive database (PRJNA745351). Any other information related to this article will be provided on request” within our cover letter.**

5. Please upload a new copy of Figures 3 and 4 as the detail is not clear. Please follow the link for more information: https://blogs.plos.org/plos/2019/06/looking-good-tips-for-creating-your-plos-figures-graphics/" https://blogs.plos.org/plos/2019/06/looking-good-tips-for-creating-your-plos-figures-graphics/

**Response: Thank you for your suggestion. We have readjusted Figures 3 and 4 to make them clearer and have uploaded them again.**

6. We note that Figure 6 in your submission contain copyrighted images. All PLOS content is published under the Creative Commons Attribution License (CC BY 4.0), which means that the manuscript, images, and Supporting Information files will be freely available online, and any third party is permitted to access, download, copy, distribute, and use these materials in any way, even commercially, with proper attribution. For more information, see our copyright guidelines: http://journals.plos.org/plosone/s/licenses-and-copyright.

**Response: Thank you for your suggestion. The picture we submitted was taken by ourselves so there was no copyright conflict.**

**Responses to comments from Reviewer #1**

The authors report transcriptome analysis in six tissues from rabbiteye blueberry ‘Brightwell’, including fully chilled leaf buds, fully chilled flower buds, early-stage flowers, full-bloom flowers, early-stage fruits (pat fruits), and “cup” fruits. Differential expressed genes (DEGs) in the paired comparisons between different tissues were identified and the potential impacts of the DEGs were discussed. The RNA-seq data presented in this manuscript are informative for a better understanding the profile of gene expressions in different tissues such as leaf buds, flower buds, flower, and fruits. Here are my major concerns (see details for 84 comments in the attached file).

Writing: Please get a professional editing service for your manuscript.

**Response: Thank you for your suggestion. We have used a professional editing service to make our manuscript more professional and accessible.**

Abstract: Please rewrite it to provide accurate information of your results.

**Response: Thank you for your suggestion. We have rewritten and enriched the abstract. Please refer to the revised text.**

Introduction: Please remove unnecessary information and provide a concise rationale for this study/report.

**Response: Thank you for your suggestion. We have removed unnecessary information and added more content related to the article as suggested.**

1.At line 50, I would suggest rewriting this paragraph by providing the following information: 1) Provide a summary of known; flowering pathways reported for both monocots and dicots; 2) Provide information on flowering mechanism in woody (especially deciduous) plants; 3) Briefly describe the reported information on flowering of vaccinium species. There have been a lot of information on vaccinium flowering reported in the past a few years.

**Response: Thank you for your suggestion.** **We have added information on pages 2–4, lines 49–86 as follows: “Flower development is the most important developmental event ... with no effect on plant flowering time in blueberry.”**

2. At line 51, What do you mean "the initial stage"? Is it bud-break (of dormancy release) after chilling? Please be aware that vernalization is not equal to chilling requirement. This is why vernalization is not often used for woody plant.

**Response: Thank you for your comment. The “initial stage” means “****at bud-break after chilling;” we have rewritten the text as suggested above.**

3. Lines 57-66, for this information, what do you want readers to know?

**Response: Thank you for your** **question. Here, we just wanted to inform the reader that some genes related to flowering have been reported previously.**

4. Line 67: Is this a ref on grape related to this study?

Response: **Thank you for your question. This was a ref on grape related to this study. However, because we didn’t describe it accurately, we have deleted it as suggested below.**

5. Line 96-97: Do you have evidence for this statement?

**Response: Thank you for your question. Unfortunately, we have not found relevant references to prove this yet.**

Results: some descriptions are not accurate. Move result descriptions from discussion to the result section.

**Response: Thank you for your suggestion. We have moved the descriptions of results from the Discussion to the Results section. Please refer to the revised content.**

6. Line 105: You need to summarize the overall RNA-seq data, for example, what is the coverage of the sequencing? Whatare the total reads? Why did you you the reference genome of highbush blueberries for your analysis of tetraploid rabbiteye blueberry?

**Response: Thank you for this reminder. We have created a S1 Table containing the overall RNA-seq data. The reference genome of highbush blueberries is the only genome of blueberry available.**

7. Line 105: When you used “false discovery rate <0.01” as a cut-off, the P-values should be all < 0.001 (if you used Edge R). But it seemed that you use P < 0.05 for some DEGs.

**Response: Thank you for this observation. We used Cuffdiff software (a part of the Cufflinks program)** **instead of edgeR to select the DEGs.** **The significance parameter was set by default.** **As you suggested, we have added this information on page 21, line 473-475 in the Materials and Methods.**

8. Line 105: Move Figure 6 here as Figure 1A to make the results easily understood.

**Response: Thank you for your suggestion. We have moved Figure 6 to the appropriate position.**

9. Line 133: Add a sentence to conclude the results of these data. The same is for the following paragraphs in this section.

**Response: Thank you for this suggestion.** **We have added the information on page 8, lines 169–173,** **as follows: “****PCA analysis of all the transcripts ... accidental mis-sampling or misidentification;”**

**and on page 7, lines 153–156, as follows: “These data showed ... transitions from buds to fruit in blueberry;”**

**and on page 10, lines 218–225, as follows: “GO analysis indicated that DEGs enriched at the early flowering ... consistent with the results of following kyoto encyclopedia of genes and genomes (KEGG) analysis;”**

**and on page 14, lines 305–307, as follows: “Changes in DEGs ... major changes during the development from bud to fruit.”**

10. Line 143: I do not think that fully chilled leaf buds will develop into flower buds and then into flower. In the other words, from leaf buds to flower buds is not a consecutive stage.

**Response: Thank you for this observation. We agree with you.** **Leaf buds to flower buds is not a consecutive stage. Some leaf buds will develop into leaves while some leaf buds will development into flower buds. This was not the focus of our study, so we just identified the DEGs between these stages in lines 184–188 and removed the rest of the related content.** **We have added an explanation on page 8,** **lines 182–184, as follows “The process from leaf buds to flower buds does not represent two consecutive stages because leaf buds will differentiate into either leaf or flower buds.”**

11. Line 147: This table can be removed by spell out the names of these seven genes in the text.

**Response: Thank you for your suggestion. We have replaced the tables with names of genes on** **lines 184–188.**

12. Line 176: This is not very consistent with what you described below. What are the criteria for being the candidate genes? I think you talked about the "DEGs of major known genes".

**Response: Thank you for your question. We have changed the title “Candidate gene involved in the flowering process” into “****DEGs related to flowering process in blueberry”** **on page 10, line 227.**

13. Line 184: What do these DEGs indicate?

**Response: Thank you for your question. The DEGs related to hormones, sugar transport, and some TF genes showed major changes in expressions during the development from buds to fruits** (**page 11, line 238).**

14. Line 189: What is floral stem? What are the DEGS (of the 34) related to flowering of flower bud formation? You need to include the DEGs in a supplemental table with gene names and expression levels.

**Response: Thank you for your suggestion. The flower stem is the part of the plant that connects the flower with the root, such as the flower stem of Arabidopsis thaliana and orchids. This term was mentioned in a number of references (e.g., Dinant, Wolff et al. 2019; doi: 10.1093/jxb/ery396). According to your suggestion, we have added S2 Table listing the DEGs (of the 34) with gene names and expression levels.**

15. Lines 200-201: DEGs of these phytohormones showed differential expressions. How do you correlate to their biological function as well as their impact on phenotype(s).

**Response: Thank you for your question. Phytohormones contribute to flower development in many ways, such as outgrowth of organs, development of male and female gametophytes and cell elongation. Ethylene can promote flowering in pineapples and other bromeliads and is involved in sex determination in hermaphrodites. It may also shorten the longevity of cut flowers. Auxin promotes cell division and growth of the plant. ABA is required for dormancy of plants and helps plants respond to stress. JA produces herbivore-induced volatiles to attract predatory insects. In our research, the expressions of genes related to auxin and JA were upregulated when transitioning from bud to flower, suggesting that the cells divided and plants prepared for attracting insects.**

**According to your suggestion, we have added information** **on page 16, lines 355–356, as follows: “Phytohormones contribute to flower development, such as outgrowth of organs, development of male and female gametophytes, and cell elongation” and on page 17, lines 377–380, as follows: “GA can promote vegetative growth to ... preparation for attracting the insects.”**

16. Line 204: What do you mean "a sizable change"?

**Response: Thank you for your question. This means many genes associated with hormones changed. We have changed “DEGs related to hormones such as auxin, GA, and JA showed a sizeable change …” into “A great many DEGs related to phytohormones such as auxin, GA, and JA showed expression changes …” to prevent confusion on pages 11–12, line 275.**

17. Lines 212-213: In your M&M, please describe how you identify and analyze these hormone-related genes? It is hard to believe that this statement "Genes related to cytokinins and auxin were up-regulated while genes related to ethylene were down-regulated". Please double check.

**Response: Thank you for your question. Some genes related to cytokinins (*cytokinin response factor 4*) and auxin (*auxin response factor 18* and *auxin efflux carrier family protein*) were up-regulated and some genes related to ethylene (*ethylene-responsive transcription factor2* and *ethylene-responsive transcription factor 4*) were down-regulated (Fig 4C).** **To prevent confusion, we have added more detail on** **page 13, lines 284–286. We identified and analyzed these hormone-related genes according to previous reports and the uniport database.**

18. Line 225: Confusing... This is a rare term.

**Response: Thank you for your concern. We have changed “flower growth” into “floral organ development and the flowering process”** **on page 14, line 302.**

19. Line 226: COL9 is similar to the CO gene that responds to photoperiod (an upstream gene of FT).

**Response: Thank you for your suggestion. Photoperiod is a factor that affects flowering; to prevent confusion, we have added “the flowering process” on page 14, line 303.**

20**.** Line 242: This figure is very confusing. Please re-label the X-axis, For example, Bud (S2/S1), Initial (S3/S2), ...... What is the inner control for your qRT-PCR?

**Response: Thank you for your suggestion. We are sorry for the confusion.** **We have added “Every sample used leaf_bud as the internal control for qRT-PCR. Actin was used as an internal control to normalize expression levels of the analyzed genes” on page 22, lines 496–497** **in the Materials and Methods. We have modified the X-axis of the figure of qRT-PCR (Fig. 5) showing that** **S2/S1, S3/S1, S4/S1, S5/S1, and S6/S1** **represent flower buds, initial flowers, bloom flowers, pad fruits, and cup fruits, respectively, and have explained it on the label** **on page 15, lines 327–328. All primers for qPCR are listed in S12 Table.**

Discussion: Please rewrite this part by moving the description of your results to the result part.

**Response: Thank you for your suggestion. We have moved the description of the results to the Results section and have added more content related to this part as suggested.**

M&M: How did you collect the tissues? What are the biological controls?

21. Line 357: How did you collect the tissues? What are the biological controls?

**Response: Thank you for your question. For your suggestion, we have added “All samples were manually picked to prevent any mechanical damage and then frozen immediately in liquid nitrogen, followed by storage at -80℃ until use. Each sample had three replicates” on page 20, lines 450–452 in the Materials and Methods.**

22. Line 372: What was the RIN?

**Response: Thank you for this reminder. We have added the information “The RIN of the 18 RNA samples was in the range from 9.0 to 9.6”** **on page 21, line 457.**

23. Line 376: Paired-end sequencing?

**Response: Thank you for your question. We are sorry this was confusing. We performed paired-end sequencing. We have added “to generate 150-bp paired-end reads”** **on page 21, line 461 in the Materials and Methods.**

24. Line 380: is Q < 20 too low? Q < 30 is often used.

**Response: Thank you for this reminder. In fact, we provided** **both Q < 20 and Q < 30 in the S1 Table. According to your suggestion, we have added the information “both Q value < 20 and Q value < 30 were used in S1 Table” on page 21, lines 465–466.**

25. Line 388: If you used FDR < 0.01, P value is < 0.001 (if you used edge R).

**Response: Thank you for your observation.** **Actually, we did not use edge R.** **The cuffdiff command, which is a part of cufflink software, was used to select the DEGs instead of edge R. We have added information as follows “Cuffdiff command, a part of cufflink software, was used to select the DEGs; therefore …” on page 21, lines 473–474 in the Materials and Methods to prevent confusion.**

26. Lines 402-403: Please include the primers in a supplemental table.

**Response: Thank you for your comment. We have added the S12 Table for primers.**

27. Lines 418-423: This is interesting. But I do not see much in your result part.

**Response: Thank you for your observation. In our research, we found no significant difference in expressions of genes related to *FT*, except *AGL20*, from bud to flower stages. Meanwhile, decreasing expression of *AGL20* indicated that the flowering process regulated by *FT* happened just before the initial flower stage. We therefore did not extensively mention this point.**

References: Please replace the unnecessary references with some more related ones. There are quite a few recent publications on blueberry flowering, blueberry genome, blueberry transcriptome analysis that are missing.

**Response: Thank you for this reminder. We have replaced unnecessary references with more related ones as suggested.**

28. Figure 6: A scale bar is needed. For the S4 flowers, were they pollinated? Were the basal parts of the flowers included for your analysis? It is not shown in this image.

**Response: Thank you for your reminder.** **We have added a scale bar in Figure 1A and we have added “The S4 flowers were almost pollinated, and the basal parts of the flowers were not included”** **on page 20, lines 449–450 in the Materials and Methods.**

**Responses to comments from Reviewer #2**

The manuscript entitled “"Transcriptome analysis of genes associated with flowering and fruit set in rabbiteye blueberry (Vaccinium ashei).” aims to unravel the molecular mechanisms of flowering and fruit set from vegetative growth to reproductive transition by evaluating the transcriptome at six developmental stages. Given the lack of RNAseq studies at the flowering stage in blueberry, this study will provide a good genomic resource for the community. However, for this study be useful, reproducible, and publishable, the authors should make the sequencing data available by depositing into public databases such as SRA, and provide more detailed results and methodology. Therefore, I would recommend “major” review and publication dependent on the availability of data and results.

Please, find my suggestions and comments below.

Title: Please, make it clear in the title that the study is for understanding the transition in developmental stages.

**Response: Thank you for your suggestion. We have changed our title “Transcriptome analysis of genes associated with 3 flowering and fruit set in rabbiteye blueberry (*Vaccinium ashei*)” to “Transcriptome analysis and identification of genes associated with floral transition and fruit development in rabbiteye blueberry (*Vaccinium ashei*).”**

Introduction: Authors should describe more about the species and how the study can be useful. Rabitteye (V. ashei) is not the main cultivated species of blueberry.

**Response: Thank you for your suggestion. Rabbiteye blueberry (*Vaccinium ashei*) is the main cultivated species in Anhui Province, which is located in the south of China. The yield of blueberry is approximately 60 thousand kilograms, bringing four hundred thousand yuan to local farms every year. Understanding the mechanism of flowering may lead to higher income for local people.**

1. P3-L67: Cite previous transcriptomic studies focused on fruit development in blueberry here.

Colle et al. 2019 (DOI: 10.1093/gigascience/giz012)

Gupta et al. 2015 (DOI: 10.1186/s13742-015-0046-9)

**Response: Thank you for your suggestion. We have added the information on page 4 lines 87–92 as follows: “Previous transcriptome analysis showed ... CG detoxification enzyme is up-regulated during fruit ripening.”**

2. P5-L96: When the authors say “Our results may be more accurate than those from previous analyses employing Trinity”, what previous analyses are you referring to?

**Response: Thank you for your suggestion. We felt this conclusion was a little arbitrary, so we have deleted it.**

3. P5-L96: Is the advantage regarding the software (Cufflinks over Trinity), or the methodology (use a reference genome over de novo assembly)?

**Response: Thank you for your question. Unfortunately, we have not found relevant references to prove this yet.**

4. P5-L103: Authors should provide a supplementary material with the differential expression results of each contrast, showing the statistical description, significance, and annotation for each gene.

**Response: Thank you for your suggestion.** **We have added S2–S6 Tables to show differentially expressed genes (DEGs) in comparisons of S2/S1, S3/S2, S4/S3, S5/S4, and S6/S5, respectively. We have also added S7–S11 Tables to show DEGs unique to each comparison.**

5. P5-L113: Colle et al. (2019) also compared pad and cup stages of berry development. A discussion comparing the results will be interesting.

**Response: Thank you for your suggestion.** **We have added a discussion on pages 18–19, lines 398–415 as follows: “There were many similar results between our research and that of Colle et al. … between the results obtained by Colle et al. and our results may be due to the sampling time.”**

6. Figure 1: Did the authors performed an all-against-all analyses to generate the Venn-diagram? It is not described in the M&M, nor results were reported.

**Response: Thank you for your reminder. We have added** **the information on page 22, lines 481–483 as follows: “****After identifying the DEGs between S2/S1, S3/S2, S4/S3, S5/S4, and S6/S5, respectively, a Venn diagram … between each stage for further analysis.”**

7. Figure2: The authors described it as “PCA of variability in DEGs”. Did you use only the DEG for the PCA construction? Please, describe in the material and methods how the PCA was performed.

**Response: Thank you for your question. We have added the information on page 22, lines 484–486 as follows: “Variation in DEGs … was studied using PCA. Samples were grouped … was performed using the R program.”**

8. P6-Line 132: Looking at the PCA clustering and the low number of genes DE between the leaf buds and flower buds, could it be a case of accidental missampling or misidentification?

**Response: Thank you for your observation. In our study, we distinguished leaf buds from flower buds only by phenotype, as shown in the following figure. This may lead to mixed samples, but this was not important in our research.**

**The upper are leaf bud and the lower are flower buds which we collected.**

9. P7-L144: Authors said that “Comparing S3 with S2, 8043 DEGs were …”. This number of DEG was not reported before.

**Response: Thank you for your comment. We are sorry that the data “8043 DEGs” was incorrect. It should have been “2298” on page 9, line 189, and this number can now be found in S3 Table.**

10. Figure 4 and 5. Please add the correspondent gene abbreviation on both plots to facilitate the comparison of results.

**Response: Thank you for your suggestion. We have added the corresponding gene abbreviation in the legend of Fig 4 on page 11-12, lines 241–258. At the same time, we have simplified the results in the manuscript.**

11. Figure 6. For me, Figure 6 should be the first figure of the paper to show the different stages being compared.

**Response: Thank you for your suggestion. We have moved Fig. 6 to Fig. 1A as suggested** **on page 7, line 144.**

12. P12-L265: The GO term “aromatic compound catabolic process” is not related to aroma compounds. It is related to any substance containing an aromatic carbon ring. Please, rewrite the discussion.

**Response: Thank you for your comment.** **We have added the information on page 16, lines 346–349 as follows: “Aromatic compound catabolic process pathways… a large number of secondary metabolites began to accumulate.”**

13. P15-L325: Do you have any other evidence that blueberries are more vulnerable to disease at the cup stage? Probably there are other hundreds of disease-related genes in the genome.

**Response: Thank you for your comment. We have added the information on page 18–19, lines 407–412 as follows: “Rabbiteye blueberry are susceptible to infection by the fungus *Exobasidium maculosum* on young fruit … may well be vulnerable to a fungal infection at the cup fruit stage” to explain this point of view.**

14. P12-L255: The reference 11 is not a transcriptomic study of flowering.

**Response: Thank you for your observation. We have deleted reference 11.**

15. P17-369: Please add a table with the sample name, number of raw reads, number of mapped reads, number of genes with reads mapped, and accession number on public databases (such as SRA).

**Response: Thank you for your reminder. According to your suggestion, we built S1 Table containing the overall RNA-seq data. The raw reads data are available in the National Center for Biotechnology Information NCBI sequence read archive database (PRJNA745351).**

16. P17-L376: Please, add more details about the RNAseq data, if paired end, read length, unstranded.

**Response: Thank you for your reminder.** **We have added the details on page 21, line 461 as follows:“to generate 150-bp paired-end reads.”**

17. P18-L378. Cite Colle et al. (2019) as the source of the genome as well.

**Response: Thank you for your suggestion. We have added the reference on page 21, line 463.**

18. P18-L384: Please clarify the differential expression analyses pipeline. Did you map the reads against the genome or assembled them? How was the tetraploid phased genome handled regarding multiple mapping reads? Was a multiple testing correction applied?

**Response: Thank you for your questions. The pipeline was as follows. We mapped the reads against the genome. The coverage of the mapping rate ranged from 51% to 74%. Cufflinks used the Benjamini-Hochberg correction for multiple-testing**.

cufflinks -p 8 -o Brightwell_bao_1_3 Brightwell_bao_1_3/accepted_hits.bam

cufflinks -p 8 -o Brightwell_bud_1-3 Brightwell_bud_1-3/accepted_hits.bam

cufflinks -p 8 -o C-cap-3 C-cap-3/accepted_hits.bam

cufflinks -p 8 -o C-pad-3 C-pad-3/accepted_hits.bam

cufflinks -p 8 -o WGC063995R WGC063995R/accepted_hits.bam

cufflinks -p 8 -o WGC063998R WGC063998R/accepted_hits.bam

cufflinks -p 8 -o Brightwell_bao_1_1 Brightwell_bao_1_1/accepted_hits.bam

cufflinks -p 8 -o Brightwell_bud_1-1 Brightwell_bud_1-1/accepted_hits.bam

cufflinks -p 8 -o C-cap-1 C-cap-1/accepted_hits.bam

cufflinks -p 8 -o C-pad-1 C-pad-1/accepted_hits.bam

cufflinks -p 8 -o WGC063996R WGC063996R/accepted_hits.bam

cufflinks -p 8 -o WGC063999R WGC063999R/accepted_hits.bam

cufflinks -p 8 -o Brightwell_bao_1_2 Brightwell_bao_1_2/accepted_hits.bam

cufflinks -p 8 -o Brightwell_bud_1-2 Brightwell_bud_1-2/accepted_hits.bam

cufflinks -p 8 -o C-cap-2 C-cap-2/accepted_hits.bam

cufflinks -p 8 -o C-pad-2 C-pad-2/accepted_hits.bam

cufflinks -p 8 -o WGC055873R WGC055873R/accepted_hits.bam

cufflinks -p 8 -o WGC063997R WGC063997R/accepted_hits.bam

cufflinks -p 8 -o WGC064000R WGC064000R/accepted_hits.bam

cuffmerge -g ../blueberry_genome/blueberry.gtf -s ../blueberry_genome/blueberryGenome.fa -p 8 assemblies.txt

cuffdiff -o diff_out -b ../blueberry_genome/blueberryGenome.fa -p 8 -L BA,BU,C,P,FB,LB,L -u merged_asm/merged.gtf ./Brightwell_bao_1_3/accepted_hits.bam, ./Brightwell_bao_1_1/accepted_hits.bam, ./Brightwell_bao_1_2/accepted_hits.bam ./Brightwell_bud_1-3/accepted_hits.bam, ./Brightwell_bud_1-1/accepted_hits.bam, ./Brightwell_bud_1-2/accepted_hits.bam ./C-cap-3/accepted_hits.bam, ./C-cap-1/accepted_hits.bam, ./C-cap-2/accepted_hits.bam./C-pad-3/accepted_hits.bam, ./C-pad-1/accepted_hits.bam, ./C-pad-2/accepted_hits.bam ./WGC063995R/accepted_hits.bam, ./WGC063997R/accepted_hits.bam, ./WGC063999R/accepted_hits.bam ./WGC063998R/accepted_hits.bam, ./WGC063996R/accepted_hits.bam, ./WGC064000R/accepted_hits.bam ./WGC055873R/accepted_hits.bam

19. P18-L390: Did you perform only GO annotation for DEG or a GO enrichment analyses? Was it performed only for unique DEGs or for all DEG? Please, clarify.

**Response: Thank you for your question.** **All the DEGs at each stage were used to perform GO enrichment analysis. According to your suggestion, we have added the details on page 22, lines 479–480 as follows: “... all the DEGs of each stage ...”**

20. P18-L397: What criteria was used to select the 9 transcripts for qRT-PCR?

**Response: Thank you for your reminder.** **We have added the details on page 22, lines 491–495 as follows:** **“There were two criteria used to select the nine transcripts for qRT-PCR: ... to make amplification easier.”**

21. P19-L401. Please, provide a table or a supplementary material with the primer sequence for each gene and control used.

**Response: Thank you for your suggestion. We have added S12 Table for primers.**
