# Supplementary material for: Transcriptome analysis and identification of genes associated with floral transition and fruit development in rabbiteye blueberry (Vaccinium ashei)
Source: PLoS One. 2021 Oct 28;16(10):e0259119. doi: 10.1371/journal.pone.0259119 (PMC8553168; doi:10.1371/journal.pone.0259119)
Supplement: Attachment — Submitted filename: Response to Reviewers_210904.docx [file pone.0259119.s014.docx]

Dear Prof. Sussmilch

Re: Manuscript reference PONE-D-21-18707R1

Please find attached a revised version of our manuscript “Transcriptome analysis and identification of genes associated with floral transition and fruit development in rabbiteye blueberry (Vaccinium ashei)”, which we would like to resubmit for publication as a research paper in *PLOS ONE.*

Your comments, as well as those of the communicating editor and reviewers, were highly insightful and enabled us to greatly improve the quality of our manuscript. In the following pages, we provide a point-by-point response to all the comments we received.

We have extensively revised the original manuscript, and the changes can be found in red color font in the text. We hope that the revisions in the manuscript and our accompanying responses will be sufficient to make our manuscript suitable for publication in *PLOS ONE.*

We look forward to hearing from you at your earliest convenience.

Yours sincerely,

Xuan Gao,

College of Life Sciences, Anhu Normal University

Wuhu, Anhui Province, China

E-mail: gaoxuan@ahnu.edu.cn

1. Filter parameters (both Q<20 and Q<30 are listed in the method and Table S1, with no explanation of which was selected to obtain “the high-quality reads” used in the analyses),

**Response: Thank you for your suggestion. We have changed information on page 23, lines 520-521 as follows: “Raw reads were filtered to obtain high-quality reads by removing low-quality reads with Q30 no less than 89.65 % (****both Q20 and Q30 were listed in S1 Table)”.**

2. Statistically significance cutoffs (p<0.05 or p<0.01? both are listed in the methods without explanation),

**Response: Thank you for your question. The statistically significant parameter was set by the software Cuffdiff. We checked again that the cutoffs are p<0.05 not 0.01. We are really sorry to make your confused. We have deleted “0.01 or” and “significant parameter set by default”** **on page 6, line 133 and have added information on page 21, line 474 as follows: “adjusted p-value <0.05”.**

3. Adjusted p-values are lacking from tables in the supporting information,

**Response: Thank you for your reminder. We have added the p value and adjusted p value (FDR) for the tables (S2 to S10) in supporting information.**

4. Methods for how the PCA and enrichment analyses were performed,

**Response:** **Thank you for your reminder. Enrichment analyses were performed by ClusterProlifer (R packages). The DEGs of each stage were generated by Cuffdiff. Org.At.tair.db were used as database. We have added information on page 22, lines 480-481 as follows: “The DEGs of each stage were generated by Cuffdiff by using database Org.At.tair.db” and the R commands were listed as follows:**

library(ggplot2)

library('org.At.tair.db')

library(clusterProfiler)

go <- enrichGO(DEGs$Entrz, OrgDb = org.At.tair.db, ont='ALL',pAdjustMethod = 'BH',pvalueCutoff = 0.05, qvalueCutoff = 0.2,keyType = 'ENTREZID')

bp<-go[go$ONTOLOGY=='BP',]

cc<-go[go$ONTOLOGY=='CC',]

mf<-go[go$ONTOLOGY=='MF',]

bp1<-bp[order(bp$Count,decreasing=T),c(3,10)][1:10,]

bp1$catelog<-rep("Biology Process",nrow(bp1))

cc1<-cc[order(cc$Count,decreasing=T),c(3,10)][1:10,]

cc1$catelog<-rep("Cellular Component",nrow(cc1))

mf1<-mf[order(mf$Count,decreasing=T),c(3,10)][1:10,]

mf1$catelog<-rep("Molecular Function",nrow(mf1))

go3<-rbind(bp1,cc1,mf1)

go3$order<-seq(1,nrow(go3),1)

go3<-na.omit(go3)

p<-ggplot(go3,aes(reorder(Description,-order),Count,fill=catelog))

p+geom_bar(stat="identity")+coord_flip()+theme(axis.title.y=element_blank(),legend.title=element_blank())

**The PCA analysis were performed by R software. All the transcripts of each sample** **analyzed by Cufflink were used as data. We have deleted “Differences in isoforms abundance between samples ... analyzed by Cufflinks”** **on page 7, line 161 and have removed this information on page 22, lines 486–488 as follows: “which was based on Fragment per Kilobase Million (FPKM)” and “analyzed by Cufflinks”. The R commands were listed as follows:**

library(ade4)

library(ggplot2)

library(RColorBrewer)

library(ggrepel)

sample<-all_transcripts[,-1]

pca<- dudi.pca(sample, scan = FALSE)

pca_eig <- (pca$eig)[1:2] / sum(pca$eig)

sample_site <- data.frame({pca$li})[1:2]

sample_site$names <- mydata$names

names(sample_site)[1:2] <- c('PCA1', 'PCA2')

sample_site$level<-mydata$level

p1 <- ggplot(sample_site, aes(PCA1, PCA2,color=level)) + theme_classic() geom_vline(xintercept = 0, color = 'gray', size = 0.4) +	geom_hline(yintercept = 0, color = 'gray', size = 0.4) + geom_point(size = 1.5)+	scale_color_manual(values = brewer.pal(6,"Set2")) + theme(panel.grid = element_line(color = 'gray', linetype = 2, size = 0.1), panel.background = element_rect(color = 'black', fill = 'transparent'), legend.title=element_blank())+ labs(x = paste('PCA1: ', round(100 * pca_eig[1], 2), '%'), y = paste('PCA2: ', round(100 * pca_eig[2], 2), '%'))

p2<-p1+geom_text_repel(aes(label=names))

5. The identity of reference genes for RT-qPCR (two different genes mentioned in the results and supporting information; how was the gene/s selected and shown to be stably expressed?).

**Response:** **Thank you for your reminder. We are really sorry to make your confused. We have checked carefully supporting information and put the two different genes (*Disease resistance protein* and *SUT4*) on the corresponding tables (S3, S6, S7 and S10). The genes we selected were based on two criteria: 1)** **The gene** **played an important role or changed considerably during flowering and fruit set. 2) The expression of genes was higher and stable in** **RNA-seq data. We may get the results easier and stable using RT-PCR in this way. We have added details on page 22, lines 494–499 as follows: “There were two criteria used to select the nine transcripts for qRT-PCR: ... to make PCR amplification easier”.**

6. There was also a concern raised about the misidentification of leaf and flower buds – this issue may affect the relevance of comparisons. Perhaps the authors could consider checking the expression of flower or leaf-specific marker genes to determine the extent of this problem and/or consider combining these samples for or removing them from comparisons? The methods do not describe how many leaf and flower buds (or other tissue types) were grouped per replicate.

**Response: Thank you for your advice. We have removed the S1 samples in all of our analysis and have added information on page 8, lines 178–182 as follows: “The process from leaf buds to flower buds does not represent ... removed S1 sample in our analysis above and below”.**

**At the same time, we remade Figure 1B, Figure 1C and Figure 5, Figure 4B and adjusted all the analysis of these figures.**

**For amount of tissue types** **per replicate, we have added information on page 20, lines 450-451 as follows: “About 200 mg of each replicaten was taken, of which 100 mg was used for RNA extraction.”.**

7. In addition, there was a concern raised that the results were overinterpreted in the discussion.

**Response: Thank you for your suggestion. We have removed “Expression of phytohormone genes ... decreased at the flower to fruit transition (SUC2 and STP14) (Fig 4C)”** **on page 18, line 497 to results** **on page 13, lines 290-294.**

**To make the paper easily accessible, we deleted “At this stage, anthocyanins ... fruit development”** **on page 18, line 497, “DEGs related to floral organ ... were all down-regulateg”** **on page 14, line 311 and “Genes encoding SAUR-like auxin-responsive protein ... the transition from bud to initial flower stage” on page 17, line 368.**

**And we have removed “NAC031 is involved in the molecular mechanisms regulating ... indicating that the flowering process was just completed at this stage” on page 19, line 416 to results on page 14, lines 304–311.**

8. There are also issues with logFC values in the latter rows of the tables in the supporting information.

**Response: Thank you for this reminder. We have added some columns including p value, adjusted p value (FDR) in the table of our supporting information (S2 to S10)**
