# Supplementary material for: Transcriptome analysis and identification of genes associated with floral transition and fruit development in rabbiteye blueberry (Vaccinium ashei)
Source: PLoS One. 2021 Oct 28;16(10):e0259119. doi: 10.1371/journal.pone.0259119 (PMC8553168; doi:10.1371/journal.pone.0259119)
Supplement: Attachment — Submitted filename: Response to Reviewers.docx [file pone.0259119.s015.docx]

1. Page 20-Line 437: Were the samples S2 used for RNA-seq analysis whole flower bud? As far as I know, blueberry flowers are inflorescence. The developmental stages of flowers on the top and bottom might be different, so the replicated samples had not gathered together.

**Response: Thank you for this observation. Yes, the whole flower bud of samples S2 were used for RNA-seq analysis.** **Considering that blueberry flowers are racemes, the developmental levels of their flowers on the top and bottom are not consistent. Therefore, in our experiments, samples are based on individual florets rather than the whole inflorescence to ensure that the developmental levels of samples collected each time were consistent. At the same time, we distinguished each sample according to their morphology. Except S1 and S2, the other four samples were easy to differentiate from. Therefore, we collected samples in the S2 developmental levels rather than the entire inflorescence. And samples in each developmental level were collected only once according to the developmental process of blueberry flowers.**

**To make the manuscript easier to understand, we have added the information on page 20 lines 448-452 as follows: “Considering that blueberry flowers are ... collected at each stage was consistent”.**

2. Page 20-Line 441: This statement about "……and the basal parts of the flowers were not included" is ambiguous. Were "the basal parts of the flowers" carpopodia or petals?

**Response: We are very sorry to make you confused. The basal parts of the flowers were the inferior ovary and sepals. To make the manuscript easier to understand,** **we have deleted the sentence: “the basal parts of the flowers were not included” on page 20 line 452, and have added the information on page 20 lines 452-453 as follows: “all parts of the S4 flower in Figure 1A were sequenced, including the inferior ovary and sepals”.**

3. Page 2-Lines 29-30, P11-Line 252, P12-Lines 267-268: "….. these DEGs were mostly enriched in phytohormone, ….". The statament is ambiguous. Please define what pathways of DEGs were enriched, biosynthesis, metabolism, or signal transduction?

**Response: We are very sorry to make you confused. To make the manuscript easier to understand, we have added the corresponding pathways on page 2 line 30, page 11 line 254, page 12 line 270, page 12 line 277, page 13 line 295, and page 14 line 312.**

4. Since the authors states that anthocyanins started to accumulate from early fruit stage. The level of anthocyanin should be measured in different development stages, at least stages S5 and S6, which is very necessary and important evidence. In fact, our unpublished data showed that the levels of anthocyanin were too low to be dected before pigments were initially accumulated in the highbush blueberry fruit peel.

**Response: Thank you for your observation. For stage S6, our previous results** **(Gao, X., et al. (2021), DOI:10.1139/CJPS-2020-0118) and Sun's results (Sun Y., et al. (2018), DOI: 10.3390 / molecules23092209) have proved that the level of anthocyanin can be detected in the green fruit (similar to stage S6). But we are very sorry that we have not found relevant references to prove that anthocyanin content can be detected in stage S5.**

**To make the manuscript more rigorous, we have deleted the sentence: “meaning that anthocyanin content started to accumulate at this stage in preparation for the abundant anthocyanin in blueberry fruits” on page 18 line 392, and have added the information with two references (Gao, X., et al. (2021), DOI:10.1139/CJPS-2020-0118; Sun Y., et al. (2018), DOI: 10.3390 / molecules23092209) on page 18 lines 392-394 as follows: “Anthocyanins may well set out to synthesize... in our previous and others’ research [38, 39]”.**

5. "The PLOS Data policy requires authors to make all data underlying the findings described in their manuscript fully available without restriction, with rare exception (please refer to the Data Availability Statement in the manuscript PDF file). The data should be provided as part of the manuscript or its supporting information, or deposited to a public repository." However, I have not found supporting information from the revised manuscript. Please deposit the sequencing data generated in this study in a suitable public repository such as the NCBI SRA database. Once the sequencing data have been deposited, please provide the information on deposition and how to access these data, including the permanent link or the unique identifier associated to it.

**Response: Thank you for your comment. In the cover letter of our previous review, we have added the following Data Availability statement: “The raw reads data are available at the National Center for Biotechnology Information NCBI sequence read archive database (PRJNA745351). Any other imformation related to this article will be provided on request”. For your suggestion, we have added the information on page 21, lines 467-468, as follows: “The raw reads data are available at the National Center for Biotechnology Information NCBI sequence read archive database (PRJNA745351)”.**

Journal Requirements:

Please review your reference list to ensure that it is complete and correct. If you have cited papers that have been retracted, please include the rationale for doing so in the manuscript text, or remove these references and replace them with relevant current references. Any changes to the reference list should be mentioned in the rebuttal letter that accompanies your revised manuscript. If you need to cite a retracted article, indicate the article’s retracted status in the References list and also include a citation and full reference for the retraction notice.

**Response: Thank you for your suggestion. We have reviewed our reference list and ensured that it is complete and correct with no retracted papers. In the revision process, we added two references (Gao, X., et al. (2021), DOI:10.1139/CJPS-2020-0118; Sun Y., et al. (2018), DOI: 10.3390 / molecules23092209) at 38 and 39.**
